# A systematic review of the relationship between severe maternal morbidity and post-traumatic stress disorder

**DOI:** 10.1186/1471-2393-12-125

**Published:** 2012-11-10

**Authors:** Marie Furuta, Jane Sandall, Debra Bick

**Affiliations:** 1 King’s College London, Florence Nightingale School of Nursing and Midwifery, James Clerk Maxwell Building, 57 Waterloo Road, London, SE1 8WA, UK; 2 King’s College London, Division of Women’s Health, Women’s Health Academic Centre KHP, North Wing, St. Thomas' Hospital, 1 Westminster Bridge Road, London, SE1 7EH, UK

**Keywords:** PTSD, Childbirth, Posttraumatic stress, Obstetric labor complication, Pregnancy complications, Puerperal disorders

## Abstract

**Background:**

The incidence of severe maternal morbidity is increasing in high-income countries as a consequence, in part, of increased obstetric intervention and increasingly complex medical needs of women who become pregnant. Access to emergency obstetric care means that for the majority of women in these countries, an experience of severe maternal morbidity is unlikely to result in loss of life. However, little is known about the subsequent impact on postnatal psychological health resulting in an evidence gap to support provision of appropriate care for these women. There has recently been increasing recognition that childbirth can be a cause of post-traumatic stress disorder (PTSD). The combination of experiencing a life-threatening complication and its management may culminate in psychological trauma. This systematic review examined the association between women’s experience of severe maternal morbidity during labour, at the time of giving birth or within the first week following birth, and PTSD and its symptoms.

**Methods:**

Relevant literature was identified through multiple databases, including MEDLINE, PsycINFO, EMBASE, CINAHL, British Nursing Index, Web of Science, Cochrane library and the British Library, using predetermined search strategies. The search terms included "post-traumatic stress disorder", "PTSD", "stress disorders, post-traumatic", "maternal morbidity", “pregnancy complications” “puerperal disorders”, "obstetric labo(u)r complication", "postpartum h(a)emorrhage", "eclampsia”. Studies identified were categorised according to pre-defined inclusion and exclusion criteria. The quality of included studies was assessed using the relevant CASP appraisal tools.

**Results:**

Eleven primary studies met review criteria. Evidence of a relationship between severe maternal morbidity and PTSD/PTSD symptoms was inconsistent and findings varied between studies. Nevertheless, there is some evidence that severe pre-eclampsia is a risk factor for PTSD and its symptoms, an association possibly mediated by other factors such as fetal/neonatal condition.

**Conclusions:**

Despite the absence of robust evidence regarding the relationship between severe maternal morbidity and PTSD/PTSD symptoms, it is crucially important that clinicians and policy makers are aware of a potential higher risk of PTSD among women who experience severe morbidity. Further studies are now needed to confirm this risk as well as to understand underlying mechanisms in order to minimise the longer term psychiatric impact of severe maternal morbidity.

## Background

Post-traumatic stress disorder (PTSD) is a condition an individual may develop in response to experiencing or witnessing a highly traumatic event. According to the Diagnostic and Statistical Manual of Mental Disorder - Fourth Edition (DSM-IV-TR) criteria for PTSD, it involves a typical subjective response such as intense fear, helplessness, or horror. Symptoms of PTSD include hyperarousal, intrusion/re-experiencing, and avoidance/numbing [1] (see Table 1). Although the concept of PTSD was initially applied to survivors of combat, rape and assault, it has increasingly been acknowledged that childbirth can be a cause of PTSD [2,3]. The prevalence following childbirth is estimated to be around 3% to 6% at around six weeks postpartum, decreasing to around 1.5% at 6 months postpartum [4]. Whether the prevalence of PTSD is higher in a postnatal population than the general population is unclear, but PTSD during the postpartum period is an important public health issue because of the longer-term negative impact of maternal mental health problems on child development [5-7] including impaired mother-infant relationship [8,9], delayed intellectual development [10,11] and psychiatric disorder in children [12]. Long-term maternal morbidity, if not identified or appropriately managed at early stage, could also increase use of health care services by women and their families [13,14]. In one US general population study, Kessler (2000) reported that costs of PTSD to society are substantial because of individual life course consequences such as childbearing issues, marital instability and work loss, the main factors influencing welfare dependency in Western societies. Kessler suggested early outreach and treatment could help to reduce the enormous burden of PTSD to individuals and society [15].

**Table 1:** **DSM-IV-TR criteria for PTSD**[1]

**A**	**Stressor**
	□ The person has experienced, witnessed, or been confronted with an event or events that involve actual or threatened death or serious injury, or a threat to the physical integrity of oneself or others.
	□ The person's response involved intense fear, helplessness, or horror
**B**	**Intrusive recollection (1 or more)**
	□ Recurrent and intrusive distressing recollections of the event, including images, thoughts, or perceptions.
	□ Recurrent distressing dreams of the event
	□ Acting or feeling as if the traumatic event were recurring
	□ Intense psychological distress at exposure to internal or external cues that symbolize or resemble an aspect of the traumatic event.
	□ Physiologic reactivity upon exposure to internal or external cues that symbolize or resemble an aspect of the traumatic event
**C**	**Avoidant/numbing (3 or more)**
	□ Efforts to avoid thoughts, feelings, or conversations associated with the trauma
	□ Efforts to avoid activities, places, or people that arouse recollections of the trauma
	□ Inability to recall an important aspect of the trauma
	□ Markedly diminished interest or participation in significant activities
	□ Feeling of detachment or estrangement from others
	□ Restricted range of affect
	□ Sense of foreshortened future
**D**	**Hyper-arousal (2 or more)**
	□ Difficulty falling or staying asleep
	□ Irritability or outbursts of anger
	□ Difficulty concentrating
	□ Hyper-vigilance
	□ Exaggerated startle response
**E**	**Duration**
	□ Duration of the disturbance (symptoms in B, C, and D) is more than one month
**F**	**Functional significance**
	□ The disturbance causes clinically significant distress or impairment in social, occupational, or other important areas of functioning

Earlier reviews of PTSD following childbirth [2-4], [16,17] identified a number of factors associated with PTSD and PTSD symptoms including pregnancy and pre-existing factors, delivery related factors and post-event environmental factors. Pregnancy and pre-existing factors include tocophobia (fear of labour), depressive symptoms during pregnancy, history of psychiatric and psychological problems, primiparity, unplanned pregnancy, trait anxiety, history of sexual trauma, low self-efficacy and perception of low support. Labour and delivery related risk factors include mode of birth (i.e. emergency caesarean, instrumental delivery), partner not present, perception of low support from partner or staff, care factors (e.g. feeling poorly informed), high fear for self and/or baby, feelings of loss of control (powerlessness), negative gap between expectation and experience of severe pain. Post-event risk factors include the absence of available postnatal support and ‘additional stress coping’ [2-4,17]. Little attention has been paid to understanding whether a woman experiencing a potentially life threatening health event during her pregnancy, labour, birth or immediate postnatal period is more likely to develop PTSD, resulting in an evidence gap to support provision of appropriate care for these women.

The primary aim of this review was therefore to assess the evidence systematically regarding a potential relationship between severe maternal morbidity occurring during pregnancy, labour and birth until the end of the first week postpartum and onset of postnatal PTSD.

### Definition, incidence and prevalence of severe maternal morbidity

'Severe maternal morbidity' (sometimes referred to as ‘near-miss’) is now used as a marker of the quality of maternity care in many counties [18,19]. These two terms are often used interchangeably for a severe, life threatening complication [20]. The term ‘near-miss’ is, however, one of the binary outcomes of life-threatening complications, as an alternative to ‘death’ and only used when a woman survives the complication, implying a positive outcome when looking at the event retrospectively [21]. Conversely, ‘severe morbidity’ can be seen as a process towards either survival or death [21]. Vais and Bewley [22] also argued the difference between a ‘near-miss’ and ‘severe maternal morbidity’, pointing out the inappropriateness of using the term ‘near miss’ to refer to the morbidity a woman actually suffers. They stated that “the term ‘near-miss’ is no longer used, as this concept was originally derived from the aviation industry and referred more to risk management than the effect on the women” (p.340) [22]. Similarly, in the WHO conceptual framework for the international classification for patient safety [23], a near-miss is more related to medical error and defined as an incident which did not reach the patient (e.g. a unit of blood being given to the wrong patient, but the error detected before transfusion commenced). When considering women’s actual experiences and the subsequent impact of obstetric complication on their psychiatric functions, it seems appropriate to use the term ‘severe maternal morbidity’ rather than ‘near-miss’.

There is no universally applicable definition of severe maternal morbidity because the severity of the condition is often determined by multiple factors such as a woman’s general health status, availability and accessibility of medical treatment, as well as human and technical resources in the healthcare system in a specific setting [21,22]. Although criteria to measure severe morbidity vary from study to study, Vais and Bewley [22] suggested that these criteria can be categorised into: 1) an organ system approach; and 2) a management or process-based approach. Say et al. [24] further categorised the organ system approach into two groups: 1) disease-specific; and 2) organ system dysfunction/failure. Using a combination of these approaches, data on fourteen major maternal morbidity outcomes are audited each month as part of the Scottish Confidential Audit of Severe Morbidity [25], including major obstetric haemorrhage, eclampsia, renal or liver dysfunction, and septicaemic shock (see Table 2). Major obstetric haemorrhage (estimated blood loss >=2500ml) has been the most frequent cause of severe morbidity in Scotland with a statistically significant and steady upward trend in incidence from 2003 to 2006 of 3.5 and 6.3 per 1000 births respectively. The rate has fallen slightly in the past 2 years, but there has been an overall increase in incidence of postpartum haemorrhage in the UK as in many other developed countries [26,27]. There is no clear cut-off to distinguish between ‘moderate’ and ‘severe’ postpartum haemorrhage. Waterstone et al. [18] used an alternative cut-off (estimated blood loss >1500ml) to estimate the incidence of severe haemorrhage in South East Thames region in England which showed an incidence of 6.7 per 1000 deliveries in 1997/1998 (see Table 2 for Waterstone et al’s criteria).

**Table 2:** Criteria and definitions of severe maternal morbidity

**Scottish Confidential Audit’s criteria and definition[**[27]**]**	
**1**	**Major obstetric haemorrhage**
	Estimated blood loss ≥ 2500ml, or transfused 5 or more units of blood or received treatment for coagulopathy
**2**	**Eclampsia**
	Seizure associated with antepartum, intrapartum or postpartum symptoms and signs of pre-eclampsia.
**3**	**Renal or liver dysfunction**
	Acute onset of biochemical disturbance, urea > 15mmol/l, creatinine > 400mmol/l, AST/ALT > 200u/l.
**4**	**Cardiac arrest**
	No detectable major pulse.
**5**	**Pulmonary oedema**
	Clinically diagnosed pulmonary oedema associated with acute breathlessness and O_2_ saturation < 95%, requiring O_2_, diuretics or ventilation.
**6**	**Acute respiratory dysfunction** Requiring intubation or ventilation for > 60 minutes (not including duration of general anaesthetic).
**7**	**Coma** Including diabetic coma. Unconscious for > 12 hours.
**8**	**Cerebro-vascular event**
	Stroke, cerebral/cerebellar haemorrhage or infarction, subarachnoid haemorrhage, dural venous sinus thrombosis.
**9**	**Status epilepticus**
	Unremitting seizures in patient with known epilepsy.
**10**	**Anaphylactic shock**
	An allergic reaction resulting in collapse with severe hypotension, difficulty breathing and swelling/rash.
**11**	**Septicaemic shock**
	Shock (systolic blood pressure < 80 mm/Hg) in association with infection. No other cause for decreased blood pressure. Pulse of 120 beats/minute or more.
**12**	**Anaesthetic problem**
	Aspiration, failed intubation, high spinal or epidural anaesthetic.
**13**	**Massive pulmonary embolism**
	Increased respiratory rate (> 20/min), tachycardia, hypotension. Diagnosed as ‘high’ probability on V/Q scan or positive spiral chest CT scan. Treated by heparin, thrombolysis or embolectomy.
**14**	**Intensive care admission/ Coronary care admission**
	Unit equipped to ventilate adults. Admission for one of the above problems or for any other reason. Include CCU admissions.
**Waterstone et al’s criteria and definition[**[18]**]**	
**1**	**Severe pre­eclampsia**
	Blood pressure 170/110 mm Hg on two occasions 4 hours apart or > 170/110 mm Hg once plus ≥ 0.3 g in 24 hours proteinuria or ≥ + + on dipstick
	OR
	Diastolic blood pressure > 90 mm Hg plus proteinuria (as above) on one occasion plus one of the following signs/symptoms: Oliguria (< 30 ml/h for 2 hours), Visual disturbances (flashing lights or blurred vision), Epigastric/right upper quadrant pain or tenderness, Thrombocytopenia (< 100x10^9^/l) Pulmonary oedema
**2**	**Eclampsia**
	Convulsions during pregnancy or in the first 10 days postpartum together with at least two of the following features within 24 hours after the convulsions: Hypertension (≥ 170/110 mm Hg), Proteinuria (≥ + on random dipstick analysis or ≥ 0.3 g in 24 hours)
	Thrombocytopenia (< 100x10^9^/l), Increased aspartate aminotransferase (≥ 42 U/l)
**3**	**HELLP syndrome**
	Haemolysis (abnormal peripheral smear or raised total bilirubin concentration (≥ 20.5 μmol/l)), raised liver enzyme activity (raised aspartate aminotransferase (≥ 70 U/l) or raised γ­glutamyltransferase (≥ 70 U/l), and low platelets (< 100x10^9^/l))
**4**	**Severe haemorrhage**
	Estimated blood loss > 1500 ml, peripartum fall in haemoglobin concentration ≥ 40 g/l or acute transfusion of 4 or more units of blood
**5**	**Severe sepsis**
	Sepsis is systemic response to infection manifested by two or more of: Temperature > 38°C or < 36°C (unless after prolonged caesarean), Heart rate > 100 beats/minute, Respiratory rate > 20/min or PaCO_2_ < 32 mmHg, White cell count > 17x10^9^/l or < 4x10^9^/l or > 10% immature forms, Plus bacteraemia (that is, positive blood cultures) or positive swab culture
	Severe sepsis is sepsis associated with one of: Organ dysfunction—for example, acute renal failure, Hypoperfusion—for example, lactic acidosis, oliguria, or acute alteration in mental state, Hypotension—that is, systolic blood pressure < 90 mm Hg or drop of > 40 mm Hg in the absence of other causes of hypotension
**6**	**Uterine rupture**
	Acute dehiscence of the uterus leading to the emergency delivery of the infant

Retrospective register-based studies in Canada, Finland and the USA have also highlighted increasing rates of severe maternal morbidity [28]. For example, a retrospective Canadian cohort study using an national database, which involved a large sample of women (n=2,548,824) who gave birth in hospitals between 1991 and 2000, observed considerable increases in the incidence of haemorrhage requiring hysterectomy (RR 1.8; 95%CI 1.5-2.1), venous thromboembolism (RR1.7; 95% CI 1.3-2.2), uterine rupture (RR1.6; 95%CI 1.4-1.8), pulmonary oedema (RR2.1; 95% CI 1.6-2.7), myocardial infarction (RR3.7; 95%CI 1.2-11.4), adult respiratory distress syndrome (RR1.5; 95% CI 1.1-2.1) and assisted ventilation (RR2.5; 95% CI 1.9-3.2) during the study period. In the same Canadian population, the presence of major pre-existing chronic disease (e.g. diabetes and heart disease) increased the risk of severe maternal morbidity 6-fold [19]. Although criteria used to measure severe maternal morbidity varied between studies, there was also a trend in the rise in the overall rate of severe maternal morbidity in the US [29] and Finland [30] showing an increase from 4.5 per 1000 births in 1991–1994 to 5.9 in 1999–2003 and from 5.9 in 1997 to 7.6 in 2002, respectively. The risk factors identified were slightly different between studies. In the US study, severe morbidity was more common at the extremes of reproductive age and among black women compared to white women, while caesarean birth (both emergency and elective) carried a significantly higher risk of life-threatening maternal complications than vaginal birth in Finland [30]; these outcomes are likely to reflect context and models of care. Changes in the demographic characteristics of women who become pregnant in Western counties are likely to lead to even higher rates of morbidity in the future as highlighted by van Roosmalen and Zwart [28] and Knight [31]. Pregnant women are more likely to be overweight or obese and many women are delaying childbirth with the potential to develop chronic health conditions needing greater medical management during pregnancy and labour, leading to increase in perinatal complications [28,31,32]. In addition to the impact of severe maternal morbidity on a woman’s physical health and well-being, it is essential to understand the magnitude of the potential impact on her mental health and well-being.

## Methods

To examine the relationship between severe maternal morbidity and postnatal PTSD, three specific review questions were developed:

1) Is there a difference in prevalence or incidence of PTSD/PTSD symptoms between women who experienced severe maternal morbidity and those who did not?

2) Is there a statistical relationship between severe maternal morbidity and PTSD/PTSD symptoms, and if so, how strong is that relationship? and

3) Does the type of severe maternal morbidity affect the relationship between severe maternal morbidity and PTSD/PTSD symptoms?

Relevant literature were identified through electronic bibliographic databases; MEDLINE, PsycINFO, EMBASE, CINAHL, British Nursing Index (BNI), Web of Science, and Cochrane library. PhD theses were searched from the British Library. The search strategy was developed in consultation with an information specialist. The search terms included "post-traumatic stress disorder", "PTSD", "stress disorders, post-traumatic", “psychological distress”, “traumatic stress” “traumatic delivery” and “birth trauma”. Although the concept of “birth trauma” includes physical injuries, birth trauma in the context of this review refers to psychological trauma as suggested by Beck [33]. Keywords related to outcomes were searched in combination with search terms related to the exposure including "maternal morbidity", “pregnancy complications” “puerperal disorders”, "obstetric labo(u)r complication", "postpartum h(a)emorrhage", “hysterectomy”, "eclampsia", "pre-eclampsia", "HELLP syndrome" and “uterine rupture”. The term "multiple organ failure" and terms for each criteria used in the Scottish Confidential Audit of Severe Maternal Morbidity (Table 2) such as "pulmonary (o)edema" and "coma" were also used in combination with the term to specify the population such as "pregnancy", “delivery, obstetric”, "labo(u)r, obstetric", "birth", “parturition”, "childbirth", "postpartum" and "postnatal". Subject headings (e.g MeSH) and free-text terms were used to maximize the sensitivity of the search. Terms were modified when necessary as each database used slightly different thesaurus terms. Restrictions were made to publications from January 1970 to August 2011 and only studies published in English were included. The year 1970 was selected because understanding of the effects of trauma on psychotic symptoms dates back to at least the 1970s [34,35] which contributed to the official introduction of PTSD into the DSM-III in 1980 [36]. All studies identified in the electronic search were first assessed for relevance by reviewing the titles, abstracts and descriptor/MeSH terms. At this stage, each study was rated as "probably relevant", "of uncertain relevance" or "irrelevant" using the inclusion/exclusion criteria listed below. Studies rated as “probably relevant" or "of uncertain relevance" were further assessed with the full texts. The electronic search was supplemented by a manual search of the reference lists in all "potentially relevant" studies. Searches were completed on Aug 2011 and updated on June 2012.

### Inclusion and exclusion criteria

The inclusion and exclusion criteria for this review are outlined in Table 3.

**Table 3:** Inclusion and exclusion criteria

**Topic**	**Inclusion criteria**	**Exclusion criteria**
**Research focus**	· The relationship between severe maternal morbidity that occurred during pregnancy until the end of the first week postpartum and the onset of PTSD/PTSD symptoms within 2 years postpartum	· Studies of PTSD/PTSD symptoms associated with miscarriage and abortion
		· Studies of PTSD/PTSD symptoms associated with medical procedure or medical intervention per se (e.g. caesarean section) without including severe maternal morbidity as a predictor of PTSD/PTSD symptoms
		· Other postnatal psychological and physical problems
		· Studies of PTSD/PTSD symptoms in pregnant women not associated with pregnancy related events but with others such as conflict, accidents or natural disasters
		· Studies examining the effects of pre-existing PTSD/PTSD symptoms on future pregnancies
**Population**	· Women who experienced (severe) maternal morbidity (eg. Major obstetric haemorrhage, pre-eclampsia/eclampsia, HELLP syndrome, admission to intensive/special care unit)	Childbearing women in general (of whom, women who experienced severe maternal morbidity not distinguishable)
**Setting/countries**	· No restriction made	· None
**Study type/design**	· Observational studies	· Descriptive studies with no comparison group
	· Experimental studies with relevant data	· Qualitative studies
	· Systematic reviews which examined the relationship between severe maternal morbidity and subsequent postnatal PTSD/PTSD symptoms	· Letter, commentary, news or short communications
		· Repeated findings originated from same study
**Language**	· English	· Non-English
**Publication**	· Published and grey literature	· None
**Time frame**	· Studies published from 1970	· Studies published before 1970

### Data extraction

Initial screening was conducted by the primary reviewer (MF). The inclusion of the studies was discussed with associate reviewers (DB and JS) until consensus was reached. To support the critical appraisal of the methodological quality of each selected study, the Critical Appraisal Skills Programme (CASP) tools were used [37]. The review process and presentation complied with the PRISMA 2009 checklist [38] (Additional file 1 Appendix 1).

## Results

**Figure 1:**
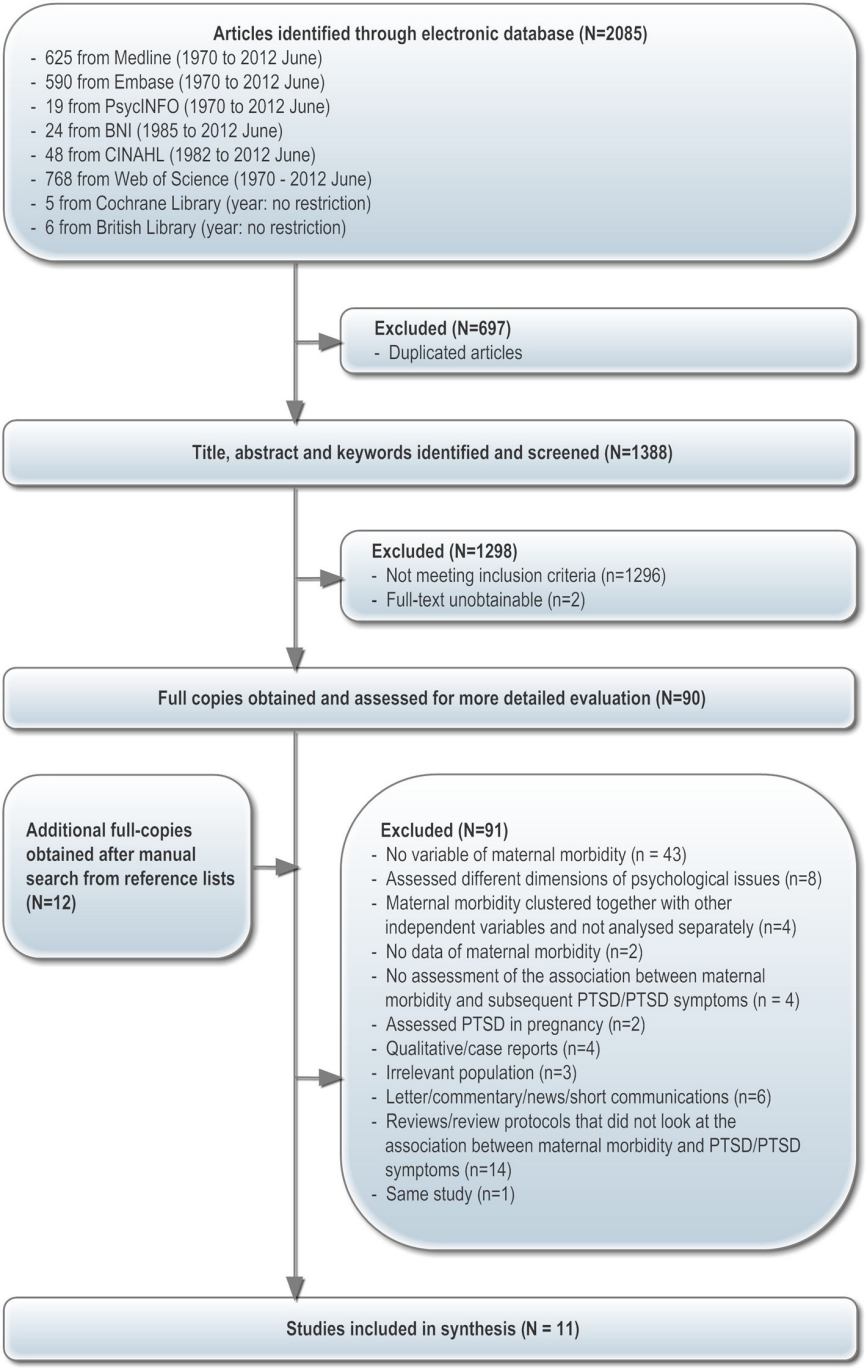
Quantitative study selection.

The search of the electronic bibliographic databases identified 2085 studies. Of these, 697 were excluded after using the bibliographic software programme, Endnote (version X4), to identify duplicate articles. Initial screening based on a review of the titles, abstracts and keywords revealed 1298 studies not relevant on the basis of inclusion/exclusion criteria (e.g. examined physical birth trauma, ineligible population) or were unobtainable (e.g. unpublished PhD thesis which were not available online). Full-text versions were obtained for the remaining 90 studies and an additional 11 studies were identified manually (total 102). After careful consideration, 90 studies were excluded. Reasons included that 1) there was no variable of maternal morbidity in analysis, 2) studies assessed different or broad dimensions of psychological and/or physical problems following maternal morbidity, 3) maternal morbidity was clustered together with other variables (e.g. socio-demographic, previous miscarriages) and not analysed separately, 4) maternal morbidity appeared to be assessed but no statistical data were provided, 5) studies reported or indicated the possibility of PTSD following maternal morbidity but the association between these two variables was not examined, 6) PTSD was assessed in pregnancy or the effects of pre-existing PTSD on pregnancy complications (e.g. miscarriage) were examined, 7) qualitative/case reports, 8) irrelevant population 9) letter, commentary, news or short communications and 10) repeated findings originated from same study (published and unpublished) with the less informative publications excluded. Fourteen systematic or narrative reviews were identified that looked at PTSD/PTSD symptoms during pregnancy or following childbirth or obstetric interventions. All studies included in these reviews were retrieved, but none provided relevant data for this review, except for the study by Ayers (1999). A total of eleven studies were included in our review. The study selection process is presented in Figure 1 and excluded studies are listed in Appendix 2 (Additional file 2).

### Overview of selected studies

The characteristics of the eleven included studies are summarised in Table 4. Studies originated from Netherlands (n=5), Australia (n=1), Canada (n=1), the UK (n=1), the US (n=1), Israel (n=1) and Nigeria (n=1). There were six prospective cohort studies [39-44], two retrospective cohort studies [45,46] and three cross-sectional cohort studies [47-49]. Four cohort studies primarily aimed to examine PTSD or PTSD symptoms following maternal morbidity or ‘difficult’ birth [40,42,44,46]. Four studies (two prospective cohorts and two cross-sectional) aimed to look at the prevalence and contributing factors related to PTSD or PTSD symptoms following childbirth in general [39,41,47,48]. Three studies (two cohorts and one cross-sectional) originally aimed to examine the effect of other exposure of interest (i.e. delivery settings, past traumatic events) or different outcomes (i.e. cognitive function) but reported relevant data for this review [43,45,49].

**Table 4:** Characteristic of the included studies

**Authors**	**Country**	**Design**	**Site**	**Size**^**†**^**(follow-up)**	**Resp. Rate**^**†**^**%**	**Time of recruit-ment**	**Criteria**		**Maternal morbidity type (Data source)**		**PTSD**		
							**Inclusion**	**Exclusion**			**Time postnatal**	**Tool**	**Admini-stration**
Adewuya et al. 2006	Nigeria	Cross-sectional	Multi. clinic. (n=5)	876	95 ^a^	Postnatal	Women attending 6 week postnatal & infant immunisation clinic	None	Hospital admission in pregnancy Manual removal of placenta	Self-report	6 wks	M.I.N.I.	Interview
Ayers 1999 (PhD thesis)	UK	Pros. cohort	Single hospital	245 (201)	70 -83 ^c^ (46–56 ^a^)	Antenatal	Gestational age 16≤, ≤ 36 wks at recruitment Good English	ElCS Poor English Other research participation Moving out No fixed address Psychiatric inpatient	Blood loss Delivery complication	Clinical records	1 week 6 weeks 6 mths	IES PSS-SR	Postal
Baecke et al. 2009	Netherlands	Retro. cohort	Single hospital	169	48-76 ^b or c^	Postnatal	Pregnancy complicated by preeclampsia and control groups	Multiple pregnancy	Pre-term preeclampsia Term preeclampsia	Clinical records	6 -18 mths	IES	Postal
Cohen et al. 2004	Canada	Pros. cohort	Multi. hospital (n=6)	198	60-87 ^b or c^	Postnatal	Age≥18 Understand English Delivered a full-term Singleton infant	Poor English Child for adoption Risk of baby (multiple infant, premature, congenanomaly, NICU, death)	maternal complications (PPH, uterine infection UTI, or retained placenta etc.)	Not clear	6-8 wks	DTS	Interview (telephone)
Creedy 1999 (PhD thesis)	Australia	Pros. cohort	Multi. hospital (n=4)	499 (141)	73 ^b^	Antenatal	Age≥18 3rd trimester pregnancy Understand English No major prenatal complication No medical problems healthy full-term infant	Risk of baby (premature, stillbirth) Pregnancy with high risk for birth complications	Delivery complication (PPH, anaemia, infection, severe post-delivery pain or manual removal of placenta etc)	Self-report	4-6 wks 3–4 mths	IES PSS-	Interview (telephone)
Engelhard et al. 2002^‡^	Netherlands	Retro. cohort	Single hospital	113	51-90 ^b^	Postnatal	Pregnancy complicated by preeclampsia and control groups Primiparas	Age<18, Illiterate in Dutch Intrauterine fetal death	Pre-term preeclampsia Term preeclampsia	Clinical records	≤ 2 yrs	PSS-SR	Postal
Hoedjes et al., 2001	Netherlands	Pros. cohort	Multi. hospital (n=4)	128 (137)	50-54 ^a or b^	Postnatal	Age≥18 Pregnancy complicated by preeclampsia speaking Dutch	--	Mild preeclampsia: Severe preeclampsia	Clinical records	6 wks 12 wks	SRIP	Postal
Lev-Wiesel et al. 2009	Israel	Pros. cohort	Single hospital	1071	96 ^c or d^	Antenatal	Women >= 5 mths pregnant at the time of recruitment	Women under psychiatric treatment	High-risk pregnancy Delivery complications (CS, preterm delivery or fetal distress etc)	Clinical records Self-report	1 mth 6 mths	PSS-I	Interview (face-to-face/ telephone)
Sorenson & Tschetter 2010	US	Cross-sectional	Commu-nity	71	75 ^c^ (53 ^b^)	Postnatal	Listed in phone book Having 'landline' phone numbers	All others who did not meet inclusion criteria	Birth complication:	Not stated	6-7 mths	PTCS	Interview (telephone)
Stramrood et al. 2010	Netherlands	Pros. cohort	Single hospital & single midwifery practice	175 (137)	71-91^c^	Antenatal	Women hospitalised with preeclampsia or PPROM	Critically ill, multiple pregnancy, A history of intrauterine fetal death, Alcohol/drug dependence Pre-existing medical conditions (eg. diabetes, hypertension, cardiovascular, renal diseases)	Preeclampsia PPROM	Clinical records	6 wks 15 mths	PSS-SR	Interview
Stramrood et al. 2011	Netherlands	Cross-sectional	Multi. Hospital (n=3) Midwifery practice (n=4)	428	47 ^a or b^	Postnatal	Women delivered 2 to 6 months prior to study with >=16 weeks of gestation	--	Pregnancy complications (pre-eclampsia, HELLP, antenatal blood loss or intrauterine death etc) Delivery complications (PPH, manual placenta removal or ICU etc)	Self-report	2-6 mths	TES-B	Web-based

Note*CS:* caesarean section; *ElCS:* elective caesarean section; *ICU:* intensive care units; *NICU:* neonatal intensive care units; *PPROM:* preterm premature rupture of membranes; *HELLP:* HELLP syndrome.

PPH: postpartum haemorrhage.

Pros: prospective; Retro: retrospective; wks: weeks; mths: months; yrs: years.

‡ Engelhard (2002) included women’s partner in their study sample, but data on women was only extracted.

† Sample size of postnatal women and response rate at postnatal period.

a) The number of all eligible women, of whom those who took part in the study.

b) The number of women who were approached, of whom questionnaire/interview were actually returned or completed.

c) The number of women who agreed to participate after the researcher approached to them, of whom questionnaires/interview were actually returned or completed.

d) Uncertain how the response rate was calculated.

### Methodological quality

The overall quality of these studies was moderate to low when assessed for the methodological quality using CASP criteria [37]. Generalisability, a lack of clear definitions of maternal morbidity and a possibility of measurement errors of PTSD/PTSD symptoms were the main issues identified from the selected studies. The methodological quality of the selected studies is summarised in Additional file 3 and discussed below. As no comparable studies were identified, and as quantitative data could not be statistically combined for a meta-analysis, extracted data were synthesised into a narrative summary. There was wide clinical heterogeneity, with different outcome measures and timing of assessment used across the included studies.

### Representativeness and generalisability

Study sample sizes ranged from 71 [48] to 1071 women [43]. The majority did not report power analysis except for Stramrood et al. [44], Creedy [41] and Hoedjes et al. [42]. The power calculation cited by Creedy [41] appeared to be performed after study recruitment, but criteria used to inform statistical significance (what difference they expected to see) was not described. Stramrood et al. [44] calculated a sample size to produce 80% statistical power for p=0.01. The study had two follow-up time points (6 weeks and 15 months postpartum). There were sufficient cases at the first follow-up point, but substantial loss to follow up in one of the study groups resulted in the sample size being smaller than that calculated for the second follow-up. Hoedjes et al. [42] discussed the possibility of low power to detect clinically meaningful differences in PTSD and related symptoms between their study groups (mild and severe pre-eclamptic women) due to the relatively low numbers of outcomes in their sample.

Response rates varied within and between studies (47% – 96%) as did the definition of the response rate. Some studies [47] defined the response rate as the number of women who entered the study from among all women who were eligible, while others defined it as the number of women who took part in the study from among those who were approached or agreed to participate at the researcher’s initial approach. In the latter cases, due to the fact that women who refused were excluded from the denominators, high response rates do not necessarily indicate good representativeness of the sample. Possible bias caused by refusal was not discussed or reported in many of the studies.

Adewuya et al. [47] recruited all women eligible (postpartum women who attended postnatal and infant immunisation clinics at 6 weeks at five government health centres in Nigeria), 95% of whom participated in the study. The study did not have any pre-specified exclusion criteria, but a few women (5%) who were critically ill, spoke a different language or refused were excluded. The study clearly described the potential bias caused by non-participants who were likely to be a high risk group resulting in possible underestimation of PTSD cases.

In a study by Lev-Wiesel et al. [43] a convenience sample of pregnant women were recruited from one hospital in Israel. Women under psychiatric care were excluded. Of the women recruited, 96% participated in a follow up interview at one and six months postpartum.

In the studies by Hoedjes et al. [42] and Stramrood et al. [49] from the Netherlands, participants were recruited from several centres (hospitals and/or midwifery practices). However, whether these sites were selected purposefully (e.g. geographical convenience) or based on pre-defined criteria was not clearly reported. Hoedjes et al. [42] approached all eligible women (whose pregnancy was complicated by pre-eclampsia), while Stramrood et al. [49] recruited a maximum of 200 women per hospital and 100 per midwifery practice to ensure ratios of delivery places were comparable with those in the Dutch population of childbearing women. Hoedjes et al. [42] clearly discussed the possibility of non-response bias. Non-native Dutch women were under-represented despite ethnicity potentially contributing to PTSD.

Stramrood et al. [44] approached pregnant women hospitalized with pre-eclampsia/HELLP (Hemolysis, Elevated Liver enzyme levels and a Low Platelet count) syndrome or preterm premature rupture of membranes (PPROM) in one university hospital. They also recruited a healthy control group with uneventful pregnancies from an independent midwifery practice. Another two studies included from the Netherlands recruited women who experienced pre-eclampsia and those who did not from one tertiary level hospital [45,46]. It was unclear whether all individuals who were eligible were actually approached or if they used the partial sample (e.g. convenient, matched). Engelhard et al. [46], Baecke et al. [45] and Stramrood et al. [44] excluded multiparous women from their samples.

Cohen et al. [40] included multiple study sites in the Toronto area of Canada but site selection criteria were not clearly reported. They also excluded multiparous women, women who could not be contacted for postpartum interview and women at risk of PTSD due to poor infant outcome (e.g. premature birth, multiple birth, admission to neonatal intensive care). The authors justified these exclusions saying "the mothers' experience with these infants would be highly stressful because of the circumstances related to the infant rather than to the experience of childbirth per se." (p.316). The authors however noted that by excluding these women, who may have been more likely to experience a difficult delivery, the extent of PTSD was probably underestimated in their study.

Ayers [39] recruited women planning normal labour and birth (ie. not booked for elective caesarean) from one hospital in England. Creedy [41] recruited women in their last trimester of pregnancy from four public hospital antenatal clinics, excluding those at high risk for obstetric problems. Women who had preterm birth or stillbirth were also excluded “due to the high probability of psychiatric morbidity following such event” (p.83). The findings from these studies are less likely to be generalisable to women with high medical risks because poor infant outcomes or elective caesarean section can be a consequence of a maternal complication.

A study from the United States [48] approached women who advertised their birth announcements in a local newspaper during a specified time period (59 days), and who had listed landline phone numbers in a publicly available phone number book. Although almost all women who gave birth at this time put their birth announcements in the newspaper (99%), the proportion of women who listed phone numbers was unclear. Many of the women (47%) contacted did not agree to participate or did not return the questionnaire.

In summary, due to a lack of clarity of reporting, assessing sample representativeness was not possible in many studies. Most studies had relatively small sample sizes and/or excluded a particular sub-group of women which could affect the generalisability of their findings.

### Exposure to maternal morbidity

In four studies, the main exposure variable was pre-eclampsia [42,44-46]. Baecke et al. [45] and Stramrood et al. [44] defined pre-eclampsia as “blood pressure exceeding 140/90 mmHg and proteinuria as urinary protein excretion over 300mg per 24h”. The same criteria were used by Engelhard et al., but in addition, they required clinical management of pre-eclampsia for at least one week. In the study by Hoedjes et al. [42], the criteria adopted by Baecke et al. [45] and Stramrood et al. [44] was used to distinguish mild from severe pre-eclampsia^a^[50]. Baecke et al. [45] and Engelhard et al. [46] did not include a separate variable for severe pre-eclampsia, but pre-eclampsia was divided into two groups, preterm pre-eclampsia and term pre-eclampsia which were used as a proxy of severity of the condition. Engelhard et al. [46] also used gestational age at admission to hospital, caesarean section and length of hospital stay as indicators of severity.

The exposure variable in the study by Cohen et al. [40] was a ‘difficult’ birth which included maternal complications (e.g., heavy bleeding after birth, uterine infection), unplanned pregnancy, perineal trauma, long labour (12 or more hours), induced labour, assisted or caesarean birth and severe labour pain. The definition of each complication was not reported.

The remaining six studies [39,41,43,47-49] assessed potential predictors of PTSD or PTSD symptoms following childbirth with no specific exposure of interest, but included variables related to maternal morbidity. Adewuya et al. [47] included hospital admission during pregnancy and manual removal of placenta. Reasons for hospital admission were not presented, but authors noted that “late detection of serious and life-threatening health problems in pregnancy could necessitate hospital admission” (p.287).

Ayers [39] included data on delivery complications and the amount of blood loss but did not state if this was estimated or measured. The type of bleeding (eg. vaginal, postpartum haemorrhage) was also uncertain. Data about other obstetric events such as infant complications, mode of birth, length of labour and use of analgesia were obtained from clinical records. However, the definition of each condition in the category of delivery complication was not given. With a high proportion of women categorised as having a delivery complication (20%), it is likely that some cases might not meet the definition of severe maternal morbidity.

Creedy [41] asked women over the telephone at 4 to 6 weeks postpartum if they experienced any maternal complications following birth (the time frame for onset was not reported). Self-reported responses included postpartum haemorrhage, medical condition (e.g. anaemia), infection (infection site not mentioned), and severe post-delivery pain. Accuracy of women’s retrospective self-report of obstetric events was checked through chart audit with a random selection of participants from one site out of four (6%, n=30) which showed the overall agreement rate was 95% [51]. Information on the item-specific accuracy was not provided. Again, considering high overall rates of self-reported maternal complications (more than 14%) among the low obstetric risk group, the majority of cases may not have been severe or life-threatening.

Stramrood et al. [49] collected information from participants using a web-based questionnaire, on pregnancy complications (e.g. pre-eclampsia/HELLP, antenatal blood loss, intrauterine death) and labour and birth complications (e.g. postpartum haemorrhage, manual placenta removal, ICU admittance).

Lev-Wiesel et al. [43] included high-risk pregnancy ‘defined as such by their gynaecologists’. The study also collected self-reported delivery complications at approximately 1 month after childbirth that included caesarean section, preterm labour, premature delivery and fetal distress. Sorenson and Tschetter [48] also included a variable of maternal birth complications, but the definition, type of complication and data source were not described.

In summary, apart from studies that primarily aimed to assess the effects of a specific type of maternal morbidity, the definition and type of maternal complication were often poorly described. Mild and more severe cases of maternal morbidity were likely to be combined. Moreover, obstetric procedures and maternal and fetal conditions tended to be pooled. Maternal morbidity in the selected studies does not necessarily comply with severe maternal morbidity as described earlier [18,27].

### Measures of PTSD

Measures of PTSD or PTSD symptoms varied. In Adewuya et al. [47], PTSD was assessed by a psychiatrist and a trained clinician using the MINI International Neuropsychiatric Interview (M.I.N.I) - a clinician administered, short structured diagnostic interview for DSM-IV and ICD-10 psychiatric disorders [52]. Creedy [41] used the PTSD Symptom Scale – Interview version (PSS-I), which supports structured clinical interview to facilitate the diagnosis of PTSD [53]. The other studies used self-report scales including the PTSD Symptom Scale – Self-report (PSS-SR) [53], the Davidson Trauma Scale (DTS) [54], the Self-rating Inventory for PTSD (SRIP) [55,56], the Traumatic Event Scale-B (TES-B) [57], the Impact of Event Scale (IES) [58] and the post-traumatic childbirth stress inventory (PTCS) [59]. Whilst the first four PTSD scales (PSS, DTS, SRIP and TES-B) follow DSM symptom criteria, the IES has less useful PTSD diagnostic utility, as it does not measure hyper-arousal, one of three dimensions of PTSD symptoms, but does provide a good indicator of PTSD [60] and is one of the most widely used screening measures for PTSD. Most scales (PSS, DTS, SRIP, IES) showed strong validity against clinical interviews following a variety of trauma events. The TES-B has been developed specifically for PTSD following childbirth and includes all DSM-IV criteria for PTSD. However, it has not yet been validated with clinical interviews [61].

Two studies [39,41] used both the PSS (either self-report or interview version) and the IES; the PSS for estimating the incidence/prevalence of PTSD following childbirth and the IES for examining predictors of PTSD symptoms. The PSS and the IES were the most frequently used scales in the current review, but as scoring systems used in each study were different, results are not comparable. The scoring methods for DTS, PSS and SRIP adopted by Cohen et al. [40], Engelhard et al. [46] and Hoedjes et al. [42] respectively were also slightly modified by researchers from the original scoring methods in order to meet DSM-IV criteria. Table 5 provides a general description of each self-report instrument and indication of the size of measurement error and likely impact on the study results.

**Table 5:** Summary of advantages and potential measurement errors of selected self-report instrument of PTSD symptoms

**Tool**	**DTS**	**IES**	**PSS-SR**	**PTCS**	**SRIP**	**TES-B**
**No. of items**	17	15	17	15	22	17
**Response scale**	5 point Likert	4 point Likert	4-point Likert	5-point Likert	4 point Likert	4 point Likert
**Validity**						
***- Sensitivity***	0.69	1.00	0.62	Not yet	0.86	Not yet
***- Specificity***	0.95(cut-off of 40on sum score)	0.78(cut-off of 19on sum score)	1.00	established	0.71	established
**Reliability**						
***- Internal consistency***	0.99	0.78 (intrusions)0.82 (avoidance)	0.91	0.93	0.90-0.94	0.84
***- Test-retest***	0.86	0.89 (intrusions) 0.79 (avoidance)	0.74	--	0.60-0.97	--
**Reporting period**	Past week	Past week	Past two weeks	Not available	Past four weeks	Past four weeks
**Specify stressor of interest**	Yes	Yes	Yes	Not available	No	Yes
**DSM-IV criteria**	B, C, D	B, C	B, C, D	Not available	B, C, D	A, B, C, D, E, F

Note: DSM-IV criteria: A = Stressor, B = Intrusion/re-experience, C = Avoidance/numbing, D = Hyperarousal, E = Duration, F = Disability. Validity and reliability were obtained from Foa et al. [53] for the PSS-SR; Davidson et al. [54] for the DTS; and Horowitz et al. [58] and Wohlfarth et al. [62] for the IES and Stramrood et al. [61] for TES-B. *The original study to test the PTCS [59] was unpublished and unobtainable.

In summary, the PSS and the DTS have high specificity (that is, the proportion of individuals classified as negative by diagnostic interview, who are correctly identified by the self-rated scale: true negative) and relatively low sensitivity (proportion of individuals classified as positives by diagnostic interview, who are correctly identified by the self-report scale: true positive). Potential measurement errors could underestimate true PTSD cases. On the other hand, the IES and the SRIP are highly sensitive and probably recognise almost all true PTSD cases [62]. However, due to relatively low specificity, potential measurement errors could lead to overestimation of the true cases, although this will depend on the cut-off used to define the cases.

As Olde et al. [4] described, the term to describe PTSD related outcomes need to be clarified as different tools measure different aspects of PTSD. From this point in the current review, the term PTSD will only be used when all diagnostic criteria of the DSM-IV-R (A: stressor; B: intrusion; C: avoidance; D: hyperarousal; E: duration and F: Disability) were met. For cases in which all symptom criteria (B, C and D) [1] were met, but some other criteria (either A, E or F) were missing, the term *PTSD-profile* will be used. The term *PTSD symptom(s)* will be used when only partial symptom criteria were met or to indicate each symptom; intrusion; avoidance or hyperarousal.

### Is there difference in prevalence/incidence of PTSD (profile/symptoms) between women who experienced severe maternal morbidity and those who did not?

Five studies [40,42,44-46] provided information on differences in the prevalence of PTSD profile or PTSD symptoms according to maternal morbidity status (Table 6).

**Table 6:** Difference in prevalence of PTSD profile/symptom (women with complication vs. women without)

**Study**	N*	**Instrument**	**Time of Assessment**	**PTSD profile & symptoms (%)**	
				**Women with complication**	**Women without (less) complication**
Baecke et al. 2009	169	IES	6 – 18 mths	PTSD symptoms	
				44%: Preterm preeclampsia	41%: Preterm, no complication
				11%: Term preeclampsia	11%: Term, uneventful
Cohen et al. 2004	198	DTS	8 – 10 wks	PTSD profile	
				0%: Maternal complication (2+)	0%: Maternal complication (0–1)
				PTS	
				59%: Maternal complication (2+)	30%: Maternal complication (0–1)
Engelhard et al. 2002	113	PSS-SR	Within 2 yrs	PTSD profile	
				28%: Preterm preeclampsia	28%: Preterm, no complication
				17%: Term preeclampsia	0%: Term, uneventful
Hoedjes et al. 2011	128	SRIP	6 wks	PTSD profile	N/A
				9%: severe & mild preeclampsia	
				11%: severe preeclampsia	
				3%: mild preeclampsia	
	137		12 wks	PTSD profile	
				5%: severe & mild preeclampsia	
				7%: severe preeclampsia	
				0%: mild preeclampsia	
Stramrood et al. 2010a	163	PSS-SR	6 wks	PTSD profile	
				11%: Preeclampsia	3% Term, uneventful
				17%: PPROM	
	137		15 mths	PTSD profile	
				11%: Preeclampsia	0% Term, uneventful
				3%: PPROM	

* The number of women included in analysis.

Hoedjes et al. [42] examined the prevalence of PTSD profile at 6 and 12 weeks postpartum among women who experienced mild (n=35) or severe pre-eclampsia (n=114). On average, the prevalence of PTSD profile (measured with the SRIP) at 6 weeks postpartum (n=128) was 9% for women who experienced either mild or severe pre-eclampsia, but the prevalence was higher for women who experienced severe pre-eclampsia (11%) than those who experienced mild pre-eclampsia (3%). At 12 weeks postpartum (n=137), the overall prevalence of PTSD profile was 5%, the prevalence for women with severe pre-eclampsia still higher (7%), compared with women with mild pre-eclampsia (0%). Hoedjes et al. [42] also examined differences in the prevalence of each PTSD symptom (intrusion, avoidance and hyperarousal) between women with mild pre-eclampsia and severe pre-eclampsia. The prevalence of each symptom was higher for women with severe pre-eclampsia than women with mild pre-eclampsia at 6 and 12 weeks postpartum.

Engelhard et al. [46] compared the prevalence of PTSD profile in two small groups of women who experienced preterm pre-eclampsia (n=18) and term pre-eclampsia (n=23), with two “control” groups, matched for gestational age at birth; preterm without any other complications (n=29) and uneventful term birth (n=43). Using the PSS-SR, 28% of women with preterm pre-eclampsia and women with preterm birth with no other complications met the PTSD profile. The corresponding figure for term pre-eclamptic women and women with uneventful term birth was 17% and 0% respectively. Chi-square tests showed that the difference in the prevalence was statistically significant between the four groups (p=0.004). More specifically, the stratified results by two groups according to gestational age at delivery (ie. the preterm and the term group) showed a difference in prevalence of PTSD profile between the two term groups (a higher prevalence in the term pre-eclampsia group than the uneventful term group), with no difference between two preterm groups (the same prevalence between preterm pre-eclampsia and preterm without complication), indicating that the association between pre-eclampsia and PTSD profile could vary depending on gestation of pregnancy at onset.

Similarly, Baecke et al. [45] assessed two major PTSD symptoms (intrusion and avoidance) using the IES with different levels of exposure; preterm pre-eclampsia (n=47), term pre-eclampsia (n=18), preterm birth but no other medical complications (n=32) and uneventful pregnancy and term delivery (n=72). A cut-off of 25 in total IES score identified that 44% of women with preterm pre-eclampsia suffered PTSD symptoms, while the prevalence was 41% for women with preterm birth but no complications, and 11% for women with both term pre-eclampsia and uneventful term delivery. The differences between the four groups were statistically significant (p < 0.001). However, stratified results by gestational age at delivery (preterm group and the term group) showed no difference in prevalence in women with and without pre-eclampsia in the same gestational age groups.

Stramrood et al. [44] compared the prevalence of PTSD profile with the PSS-SR, at 6 weeks (t_1_) and 15 months (t_2_) postpartum in three groups; pre-eclampsia/HELLP (t_1_: n=57, t_2_: n=44), preterm premature rupture of membranes (PPROM) (t_1_: n=53; t_2_: n=31) and term uneventful pregnancy (t_1_: n=65; t_2_: n=62). The prevalence of PTSD profile was found to be 11% among women with pre-eclampsia/HELLP and 17% for women with PPROM at 6 weeks postpartum, which was significantly higher than following uneventful pregnancies in the control group (3%) (p=0.04). Stramrood et al’s [44] sample included women whose babies died (n=12). When these women were excluded from analysis, the difference between groups (pre-eclampsia/HELLP and PPROM vs. uneventful term groups) was no longer significant at 6 weeks postpartum (p=0.06) indicating that the death of the baby could have a mediating role. At 15 months postpartum, 11% of women with pre-eclampsia/HELLP met the PTSD profile criteria, compared with no controls. The study noted that the low response rate in the PPROM group at 15 months postpartum did not permit any firm conclusions.

Cohen et al. [40] examined the prevalence of PTSD profile among new mothers with a full term singleton infant, using the DTS. In a sample of 200 women, 22 experienced two or more maternal complications and 176 experienced none or one maternal complication during pregnancy and delivery (e.g., heavy bleeding after birth, uterine infection, urinary tract infection, retained placenta). At 8–10 weeks following the birth, telephone interviews with the women revealed that no study participants met their predefined study criteria for PTSD-profile. The prevalence of the ‘high postpartum stress’ was however high among women who had two more maternal complications (59.1%) compared to women who had none or one complication (29.6%). The difference was statistically significant using chi-square test (p= 0.005), but the results should be interpreted with a caution as this dichotomous outcome category (high vs. low postnatal stress) was based on the authors developed a scoring method using the DTS.

In summary, the estimated prevalence of PTSD profile and PTSD symptoms measured by self-rated scales in selected studies varied from 0% to 44% following maternal morbidity. Confidence intervals for prevalence were not provided for any of studies, but the wide range of prevalence can be explained by the small sample size in each study. High prevalence of PTSD symptoms (11-44% at 6–18 months postnatal) in the study by Baecke et al. [45] may be due to the lower specificity produced by the cut-off of total IES score (total IES>25) which was selected to define the cases. However, the results of remaining studies indicated that an experience of maternal morbidity, especially of severe or preterm pre-eclampsia could have potentially increased the prevalence of PTSD profile and PTSD symptoms during postpartum period.

What we know

The prevalence of PTSD profile and PTSD symptoms up to 2 years postpartum is potentially higher among women who experienced maternal morbidity, especially severe morbidity and/or had a preterm birth (these two are often linked)

What we don’t know

There is no evidence of whether women who experienced severe maternal morbidity are more likely to develop diagnostic PTSD (e.g., meet DSM-IV criteria)

### Is there a statistical relationship between severe maternal morbidity and PTSD (profile/symptoms), and if so, how strong is that relationship?

Five out of the eleven studies examined factors contributing to the presence of PTSD or PTSD profile/symptoms but treated the outcome as a dichotomous variable (eg. presence or absence of PTSD), while six studies examined contributors to the severity of PTSD symptoms by treating the outcome as a continuous variable (ie. total score of self-administered measurements for PTSD symptoms).

Hoedjes et al. [42] conducted logistic regression analyses for each predictive variable, and showed that the PTSD profile and PTSD symptoms at 6 and 12 weeks postpartum were more frequently present among women who had severe pre-eclampsia than women with mild pre-eclampsia. The prevalence was also higher among younger women, women who had severe pre-eclampsia, who were delivered by caesarean section, who had a lower gestational age at delivery, a lower birth weight, and among women whose child had been admitted to the neonatal intensive care unit or had died. These variables however were not adjusted for each other. Unadjusted (crude) odds ratio (OR) and statistical significance for each predictors are presented in Table 7.

**Table 7:** Association and effect size of maternal morbidity and other variables on PTSD (profile/symptoms)

**Study**	**N**	**Method**				**Results (in case of ORs: risk vs. reference)**			
Adewuya et al., 2006	876	Stepwise multiple regressions	M.I.N.I	PTSD	6 wks	Admission due to pregnancy complication: yes vs. no	Adjusted OR: 11.9 ^†^		(95%CI: 6.4–22.1)
						Mode of delivery			
						- Instrumental vs. spontaneous vaginal	Adjusted OR: 7.9 ^†^		(95%CI: 3.9–16.2)
						- EmCS vs. spontaneous vaginal	Adjusted OR: 7.3 ^†^		(95%CI:3.5–15.2)
						- ElCS vs. spontaneous vaginal	Adjusted OR: 2.0		(95%CI: 0.4–8.9)
						Mode of placental removal: manual vs. normal	Adjusted OR: 5.0 ^†^		(95%CI: 2.4–10.1)
						Perceived control in childbirth: LAS < 40 vs. > 40	Adjusted OR: 5.1 ^†^		(95%CI: 2.7–9.5)
Ayers, 1999	220	Mann Whitney Spearman's correlation	IES	Intrusions (sub-sum score)	6 wks	Delivery complication: presence vs. absence	ns		
						Amount of blood loss	ns		
		Kruskal-Wallis				Type of delivery (eg. EmCS)	ns		
		Partial correlation (removing an effect of PTSD symptoms in pregnancy)				Appraising birth as traumatic	Partial correlation β=.20 ** (one tailed)		
						Different from how women wanted to be	Partial correlation β=.17*		
	201				6 mths	Delivery complication: presence vs. absence	ns		
						Amount of blood loss	ns		
						Type of delivery (eg. EmCS)	ns		
						Appraising birth as traumatic	Partial correlation β=.19 ** (one tailed)		
						Different from how women wanted to be	Partial correlation β=.22**		
	220			Avoidance (sub-sum score)	6 wks	Delivery complication: presence vs. absence	ns		
						Amount of blood loss	ns		
						Type of delivery (eg. EmCS)	ns		
						Appraising birth as traumatic	Partial correlation β=.23 ** (one tailed)		
						Different from how women wanted to be	Partial correlation β=.35***		
						
	201				6 mths	Delivery complication: presence vs. absence	Unadjusted U=2553 *		
						Amount of blood loss	ns		
						Type of delivery (eg. EmCS)	ns		
						Appraising birth as traumatic	Partial correlation β=.24 *** (one tailed)		
						Different from how women wanted to be	Partial correlation β=.29 ***		
Baecke et.al, 2009	169	Method for ORs: not stated	IES	PTSD symptoms	6-18 mths	Preterm preeclampsia vs. Term, uneventful	(Adjusted?) OR: 6.2^†^	(95%CI: 2.5-15.8)	
						Preterm preeclampsia vs. Term preeclampsia	(Adjusted?) OR: 6.2^†^	(95%CI: 1.3-30.1)	
						Preterm, no complication vs. Term, uneventful	(Adjusted?) OR: 5.5^†^	(95%CI: 2.0-15.2)	
Cohen et al., 2004	184	Multivariable logistic regression	DTS	Postpartum stress (high/low)	8-10 wks	Maternal complications: 2+ vs. 0-1	Adjusted OR: 4.0^†^	(95%CI: 1.3-12.8)	
						Depression during pregnancy: yes vs. no	Adjusted OR: 18.9^†^	(95%CI: 5.8-62.4)	
						History of traumatic events: 2+ vs. 0–1	Adjusted OR: 3.2^†^	(95%CI: 1.2-8.3)	
						Born in Canada vs. Not born in Canada	Adjusted OR: 3.2^†^	(95%CI: 1.3-8.1)	
						Income (Canadian $)			
						- lowest (<$32,000) vs. high (>$8000)	Adjusted OR: 0.1^†^	(95%CI: 0.02-0.5)	
						- middle ($32,000-80,000) vs. high (>$8000)	Adjusted OR: 0.4^†^	(95%CI: 0.2-0.8)	
Creedy et al., 1999	499	Simple regression	IES	PTSD symptom severity (sum score)	4-6 wks	Preparedness for labour and delivery	ns		
		Stepwise multiple regressions				Maternal complications	ns		
						EmCS	Adjusted β=.20***		
						Forceps delivery	Adjusted β=.17***		
						Vacuum delivery	Adjusted β=.14**		
						Post-delivery pain	Adjusted β=.16***		
						Neonatal complications	Adjusted β=.10*		
		Hierarchical multiple regression				Final model (Accounted for 21% of variance)			
						Perception of maternity care (step 1)	Adjusted β=.-32***		
						Obstetric intervention (step 2)	Adjusted β=.26***		
						
	141	Multiple regression			3 mths	Final model (Accounted for 24% variance)			
						Preparedness for labour and delivery	Adjusted β=.-16*		
						Obstetric intervention	Adjusted β=.15*		
						Perception of maternity care	Adjusted β=.42***		
Engelhard et.al, 2002	113	Hierarchical multiple regression	PSS-SR	PTSD symptom severity	Within 2 yrs	Final mode (Accounted for 61% of the variance)			
						Gestational age at admission (step 1)	ns		
						Peritraumatic distress (step 2)	ns		
						Peritraumatic dissociation (step 2)	Adjusted β=.27**		
						Negative interpretations (step 3)	Adjusted β=−.40*		
						Thought suppression (step 3)	Adjusted β=−.25*		
Hoedjes et al., 2011	149	Logistic regression for each variable (adjusting only for assessment time – 6 and 12 weeks postpartum using GEE^‡^)	SRIP	PTSD profile (yes/no)	6-12 wks	Severity of preeclampsia: severe vs. mild	Unadjusted OR: 5.0*	(95%CI: 0.6–38.8)	
						Mode of delivery: CS vs. vaginal	Unadjusted OR: 8.4*	(95%CI: 1.1–65.5)	
						Age	Unadjusted OR: 0.6*	(95%CI: 0.4–0.7)	
						Gestational age at delivery	Unadjusted OR: 0.8*	(95%CI: 0.7–1.0)	
				Intrusions (yes/no)		Severity of preeclampsia: severe vs. mild	Unadjusted OR: 5.5*	(95%CI: 1.6–18.7)	
						Mode of delivery: CS vs. vaginal	Unadjusted OR: 4.3*	(95%CI: 1.7–10.6)	
						Admission to NICU: yes vs. no	Unadjusted OR: 5.9*	(95%CI: 2.4–15.0)	
						Perinatal death: yes vs. no	Unadjusted OR: 7.1*	(95%CI: 1.8–27.8)	
						Age	Unadjusted OR: 0.8*	(95%CI: 0.7–0.9)	
						Gestational age at delivery	Unadjusted OR: 0.9*	(95%CI: 0.8–0.9)	
						Birth weight	Unadjusted OR: 0.5*	(95%CI: 0.3–0.8)	
				Avoidance (yes/no)		Mode of delivery: CS vs. vaginal	Unadjusted OR: 3.9*	(95%CI: 1.1–13.9)	
						Admission to NICU: yes vs. no	Unadjusted OR: 4.3*	(95%CI: 1.2–15.6)	
						Age	Unadjusted OR: 0.7*	(95%CI: 0.6–0.8)	
						Gestational age at delivery	Unadjusted OR: 0.9*	(95%CI: 0.8–0.9)	
						Birth weight	Unadjusted OR: 0.4*	(95%CI: 0.2–1.0)	
						
				Hyperarousal (yes/no)		Severity of preeclampsia: severe vs. mild	Unadjusted OR: 3.0*	(95%CI: 1.2–7.9)	
						Mode of delivery: CS vs. vaginal	Unadjusted OR: 2.6*	(95%CI: 1.2–5.7)	
						Admission to NICU: yes vs. no	Unadjusted OR: 2.8*	(95%CI: 1.3–5.8)	
						Perinatal death: yes vs. no	Unadjusted OR: 6.6*	(95%CI: 1.1–39.6)	
						Age	Unadjusted OR: 0.9*	(95%CI: 0.8–1.0)	
						Gestational age at delivery	Unadjusted OR: 0.9*	(95%CI: 0.8–1.0)	
						Birth weight	Unadjusted OR: 0.6*	(95%CI: 0.4–0.8)	
Lev-Wiesel et al, 2009	1071	Linear multiple regression	PSS-I	PTSD symptoms severity	6 mths	Final model (Accounted for 41% of the variance)			
				(sum score)		Subjective pain and distress during delivery	Adjusted β=.51***		
						PTS during pregnancy	Adjusted β=.04		
						Delivery complications	Adjusted β=.04		
						Depression during pregnancy	Adjusted β=.15***		
						History of traumatic events	Adjusted β=.08**		
						High risk pregnancy	Adjusted β=.03		
Sorenson & Tschetter, 2010	71	Point-biserial correlation coefficient	PTCS	Posttraumatic childbirth stress (low/high)	6–7 mths	Maternal complications: yes vs. no	Unadjusted rpbs = 0.28 ^†^		
						Infant complications: yes vs. no	Unadjusted rpbs = 0.25 ^†^		
Stramrood et al, 2010	175	Hierarchical multiple regression	PSS-SR	PTSD symptoms severity	6 wks	Final model (Accounted for 39% of the variance)			
						A history of depression (step 1)	Adjusted β=.23**		
						BDI scores during pregnancy (step 1)	Adjusted β=.33***		
						Death of infant (step 2)	Adjusted β=.29***		
						Hospital admission of the infant (step 2)	ns		
						Birth weight (step 2)	ns		
						Diagnosis of the mother (PE vs PPROM) (step 2)	ns		
						CS (step 2)	ns		
						
Stramrood et al, 2011	428	Hierarchical multiple regression	TES-B	PTSD symptoms severity (sum score)	2 to 6 mths	Final model (Accounted for 41% of the variance)			
						Country of origin (step 1)	Adjusted β=.004		
						Primiparity (step 1)	Adjusted β=.06		
						Preeclampsia/HELLP syndrome (step 1)	Adjusted β=.08		
						Hypertension (step 1)	Adjusted β=.04		
						Preterm delivery (step 1)	Adjusted β=.04		
						Secondary/tertiary care (step 2)	Adjusted β=−.09		
						Hospital delivery (step 2)	Adjusted β=−.05		
						Induction of labour (step 2)	Adjusted β=−.02		
						Instrumental vaginal delivery (step 2)	Adjusted β=−.08		
						Unplanned caesarean section (step 2)	Adjusted β=.11**		
						Postpartum haemorrhage (>1L) (step 2)	Adjusted β=.06		
						Manual placenta removal (step 2)	Adjusted β=.04		
						Perinatal death (step 2)	Adjusted β=.06		
						N(I)CU admittance (infant) (step 2)	Adjusted β=.05		
						ICU admittance (mother) (step 2)	Adjusted β=.03		
						Fear of childbirth (high) (step 3)	Adjusted β=.02		
						Delivery worse than expected (step 3)	Adjusted β=.01		
						Intensity of pain (high) (step 3)	Adjusted β=.11*		
						Sense of Coherence (low) (step 3)	Adjusted β=.53***		

Note *p<0.05. **p<0.01, ***p<0.001, ns: none significance, † significance but the level of significance was not reported.

‡ GEE: generalized estimating equation.

Baecke et al. [45] reported that preterm pre-eclamptic women had 6.2 times higher odds of having PTSD symptoms than women who had uneventful term delivery. They had also 6.2 times higher odds of PTSD symptoms than women who had term pre-eclampsia, but with a very wide confidence interval (95% CI: 1.3-30.1). In addition, it was not clear if findings were adjusted for potential confounders as the statistical methods used to provide the odds ratio were not described.

Adewuya et al. [47] conducted a stepwise regression analysis followed by bivariate analysis to identify predictors of PTSD in Nigerian women at 6 weeks postpartum. The results showed the most significant predictors of PTSD were pregnancy-related hospital admission, instrumental delivery, and emergency caesarean section (but not elective), loss of control during childbirth (as measured by the 10-item Labour Agentry Scale at 6 weeks) and manual removal of placenta.

Multivariable logistic regression conducted by Cohen et al. [40] found that women with two or more maternal complications were more likely to have high level of postpartum stress than women with fewer complications after controlling for the effects of other variables (e.g. depression during pregnancy and history of traumatic events) (adjusted OR=4.0; 95%CI=1.3-12.8). The strongest predictor of high postpartum stress was depression during pregnancy, but with a very wide confidence interval (adjusted OR=18.9, 95%CI=5.8-62.4). A history of two or more traumatic life events, ‘born in Canada’ (native Canadian) and higher income had also high odds of having high postpartum stress. The latter two were unexpected findings for the authors who considered “women from developed countries may be more likely to admit to having such symptoms than women from other cultures” (p. 323).

In a sample of women who experienced pre-eclampsia (both preterm and term) and preterm delivery without complication, Engelhard et al. [46] developed a three-step hierarchical multiple regression model to test the relative contribution of predictive variables that were statistically correlated with severity of PTSD symptoms in their bivariate analysis. In the first step, the gestational age of pregnancy on admission was entered in the model which alone accounted for 7% of the variance in severity of PTSD symptoms. On the second step, peri-traumatic reactions (distress and dissociation) were added into the model which accounted for 43% of the variance. After adjusting for these variables, the association between PTSD symptoms and gestational age was no longer statistically significant. On the final model (the third step), individual psychological characteristics were added: peri-traumatic dissociation (β=.27, P=0.008); negative interpretations of symptoms (β=.40, P < 0.001); and thought suppression (β =.25, P=0.012) which together accounted for 61% of PTSD symptoms among women participants (F=34.84, P=0.001). However, all of these psychological characteristics were “based on the subjects’ recall of how they felt up to two years previously”, and the possibility of recall bias cannot be discounted (p. 263) [46]. Caesarean section and length of hospital stay (used as indicators of severity of pregnancy complication) were not entered in the model these variables were not statistically correlated with severity of PTSD symptoms (CS: r=.22, p=0.07, length of hospital stay: r=.19, p=0.12). Stramrood et al. [44] performed two-step hierarchical multiple regression analyses to assess factors related to the severity (sum-score) of post-traumatic stress symptoms at 6 weeks postpartum. Variables entered in the first step were history of depression (yes/no) and Beck Depression Inventory (BDI) scores during pregnancy which accounted for 29% of the variance. In the second step, variables indicative of the well-being of both mother and infant were added, that is, death of infant, hospital admission of the infant, birth weight, diagnosis of the mother (pre-eclampsia vs PPROM) and caesarean delivery which accounted for 39% of the variance. Risk factors that remained statistically significant after controlling for the effects of each variable were self-reported history of depression (β=.23, P=0.007), a high BDI score during hospitalization (β =.33, P=0.001), and infant death in the postpartum period (β=.29, P=0.001).

Similarly, a three step hierarchical multiple regression model in Stramrood et al. [49] showed significant predictors of severity of post-traumatic stress symptoms (the TES-B sum-scores) at 2 to 6 months were unplanned caesarean section (β=.11, P < 0.01), high intensity of pain (β=.11, P < 0.05), and low sense of coherence (β=.53, P < 0.001) which explained 41% of the variance in post-traumatic stress symptoms at 2 to 6 months. Initial differences, which were found with non-parametric bivariate analysis in post-traumatic stress symptoms between women who experienced postpartum haemorrhage (>1000 ml) or pre-eclampsia/HELLP and those who did not, disappeared after controlling for the effects of each variable (e.g. mode of delivery).

Ayers [39] examined factors associated with PTSD symptoms, intrusion and avoidance, in a cohort of women in the UK at three time points postpartum; 1 week (n=245); 6 weeks (n=220); and 6 months (n=201). The study identified women who had severe PTSD symptoms in pregnancy (n=18, as measured with the MMPI-2-PTSD scale) and controlled for the effect during analysis. Using non-parametric statistical tests, the study found factors strongly correlated with avoidance at all three points were subjective birth experience as measured at one week postpartum (the absence of positive emotions, appraising birth as traumatic, lack of control over analgesia and different from how women wanted it to be). On the other hand, key factors correlated with intrusions over 6 months postpartum included pre-existing belief and anxiety. Interestingly, maternal complications had a negative association with PTSD symptoms - women with no labour or birth complications had statistically significantly higher symptoms of intrusion at one week after birth (Mann Whitney, U=2619.5, p < 0.05) and higher symptoms of avoidance at six months postpartum than women who did (Mann Whitney, U=2553, p < 0.05). There was no statistical relationship between type of delivery (eg. emergency caesarean section), type of labour onset or complication with the baby and PTSD symptoms (intrusion or avoidance). Spearman's rank correlation coefficient also demonstrated no statistical correlation between the amount of blood loss and either intrusion or avoidance. Blood loss, although initially correlated with women’s self-appraisals of their birth as traumatic as measured at 1 week after birth using a 10 cm visual analogue response scale (Spearman’s ρ .29, p < 0.001), was not significant after controlling for negative emotions during birth, lack of positive emotion in birth and mode of delivery. Only the key results relevant to this study are presented in Table 7.

Simple regression and stepwise multiple regression analysis conducted by Creedy [41] revealed that neither maternal delivery complications (self-reported at 4–6 weeks after giving birth) nor antenatal variables (i.e. preparedness, obstetric risk, likelihood of unexpected events, anticipatory anxiety, level of partner support, and state anxiety) were predictive of PTSD symptoms (the IES total score) at 4–6 weeks among women in Australia (n=499) who had a term delivery with no serious risk of obstetric complication during pregnancy (figures not presented for maternal complication). Factors associated with PTSD symptoms were women’s retrospective self-report of obstetric intervention (β=.35, P < 0.001) which looked at the cumulated impact of five key variables (ie. emergency caesarean section (β=.20, P < 0.0001), forceps delivery (β=.17, P < 0.0001), post-delivery pain (β=.16, P < 0.0001), vacuum delivery (β=.14, P < 0.002) and diagnosis for the baby – a congenital condition or some other medical complication on delivery (β=.10, P < 0.02)). The perception of maternity care (measured at 4–6 weeks postpartum) also had a strong negative association with PTSD symptoms (β=−.39, p < 0.001) indicating lower the perception of maternity care, the higher the risk of PTSD symptoms. The study further developed hierarchical regression models to determine whether the relationship between obstetric intervention and PTSD symptoms at 4–6 weeks postpartum was mediated by perception of care. The model identified that perception of care was not a mediator but had an additive effect on the PTSD symptoms; in other words, both obstetric intervention (β=.26, P < 0.001) and perception of care (β=.32, P < 0.001) directly contributed to the outcome. Creedy also examined contributors to PTSD symptoms at 3 months postpartum (n=141) among women who described a stressful birth event and had reported at least three trauma symptoms at 4–6 weeks using the IES. Multiple regression analyses showed that level of preparedness for labour and delivery (as measured in pregnancy by a 5 point Likert scale self-assessment question ‘how well prepared do you feel for childbirth?’) (β=.-16, P=0.03), the perception of intrapartum care (β=.42, P=0.0001) and obstetric intervention (β=.15, P < 0.05) were associated with PTSD symptoms at 3 months postpartum that accounted for 24.5% of variance. None of specific obstetric intervention (e.g. emergency caesarean section, forceps delivery) was statistically associated with PTSD symptoms at this time point.

Linear regression models in Lev-Wiesel et al. [43] showed that neither delivery complications nor high risk pregnancy were statistically associated with PTS symptoms (PSS-I total score) at 6 months after delivery among 1071 women in Israel. Instead, higher levels of subjective pain and distress during delivery assessed at 1 month after delivery (β=.51, p < 0.001), depression during pregnancy (β=.15, p < 0.001) and history of life traumatic events (β=.08, p < 0.01) were found to be predict variables of PTS symptoms.

Sorenson and Tschetter [48] reported a positive correlation between maternal complications and perinatal trauma symptoms (yes/no) measured at 6–7 months postpartum using the author developed measurement (point-biserial correlation coefficient: rpbs = 0.28).

In summary, results for the relationship between severe maternal morbidity and PTSD (profile/symptoms) from selected studies were inconsistent. This could be explained by the following factors: selection bias due to a lack of definition of maternal morbidity, unreliable data sources, the sample only included relatively healthy women (e.g. term delivery), or data unadjusted for potential confounders. However, four studies [42,44-46] which had clear definitions of maternal morbidity and reliable data sources tended to indicate that severe maternal morbidity could potentially increase the risk of postpartum PTSD symptoms. Of these, three studies conducted analysis only in a sample of patients with pre-eclampsia/PPROM or preterm delivery without including medically uncomplicated women [42,44,46]. The results indicated that the association between maternal morbidity and PTSD symptoms may not be direct but possibly mediated by other factors such as distress and/or neonatal conditions (e.g. prematurity, death). However, due to the small sample size of these studies (n < 180), definite conclusions cannot be drawn.

What we know

The available evidence about the relationship between maternal morbidity and PTSD/PTSD symptoms is inconsistentand varies between studies

The relationship is possibly mediated by other factors such as fetal/neonatal conditions (e.g. prematurity, death) andperitraumatic dissociation.

What we don’t know

There is no robust evidence to show whether there is a direct relationship between severe maternal morbidity and PTSD/PTSD symptoms after controlling for other predictors and potential confounders (e.g. mode of delivery, pre-existing psychological morbidity)

Does the type of severe maternal morbidity affect the relationship between severe maternal morbidity and PTSD (profile/symptoms)?

Only five studies examined a specific maternal complication; pre-eclampsia [42,44-46] and blood loss [39]. As described earlier, pre-eclampsia, particularly severe pre-eclampsia and preterm pre-eclampsia increased PTSD profile or PTSD symptoms postpartum, while no correlation was found between the amount of blood loss and PTSD symptoms [39]. In Ayers' study, the range of blood loss was not reported, and it is uncertain if there were any cases of severe obstetric haemorrhage. Postpartum haemorrhage was examined by Cohen et al. [40] and Creedy [41], but it was clustered together with other complications (e.g. urinary tract infection, site unspecific infection). In summary, from evidence currently available, this question cannot be answered.

What we know

There is some evidence that pre-eclampsia might be linked to PTSD profile/PTSD symptoms

There is insufficient evidence to inform a relationship between obstetric haemorrhage and PTSD profile/PTSD symptoms

What we don’t know

There is no evidence to determine whether the type of severe maternal morbidity affects the relationship between severe maternal morbidity and PTSD/PTSD symptoms

## Discussion

This paper describes a systematic review of the association between women experiencing severe maternal morbidity during labour, at the time of giving birth or within the first week following birth, and post-traumatic stress disorder. Findings are based on a comprehensive literature search and rigorous critical appraisal of included studies.

No high quality quantitative studies were identified to determine whether women who experienced severe maternal morbidity are more likely to develop PTSD or traumatic stress symptoms than women who did not. Our review however found a potential higher risk of PTSD following severe maternal morbidity. The prevalence of PTSD profile among pre-eclamptic women from 6 weeks up to two years postpartum was 5%-44%. This appeared to be a higher percentage than that found in an earlier systematic review on PTSD following childbirth in general. For example, Olde et al. [4] found that the prevalence of PTSD among mothers who had successful birth outcomes (including normal births and births by caesarean section, but excluding pregnancy complications) was estimated to be approximately 3% to 6% at around six weeks postpartum, decreasing to around 2% at six months postpartum. Similarly, a narrative review by Ayers [63] suggested a prevalence of 0%-7% of PTSD within one year after giving birth, while the figure was higher for at-risk groups (i.e., premature birth or stillbirth), up to 26% at one month postpartum. These are the estimates from different populations, but provide some idea that the rate may also be higher for women who experienced severe maternal morbidity.

An earlier systematic review by Tedstone and Tarrierb [64] on PTSD following other medical illnesses (e.g., myocardial infarction, acute lung injury and stroke) suggested that the link between the severity of the illness and the development of PTSD is not always straightforward. Recent prospective studies in low-income countries [65] showed that the development of psychological distress following severe maternal morbidity was mediated by perinatal loss. Our review also identified the possibility of an indirect relationship in which the association between maternal morbidity (i.e. pre-eclampsia) and post-traumatic stress symptoms differed according to a third factor such as gestational age at delivery, baby’s condition (e.g. prematurity, death) and negative interpretations of symptoms. However, due to the methodological limitations in selected studies, possible pathways towards PTSD or mechanisms underlying the relationship could not be fully explained. Insufficient evidence was available to compare the outcomes following different types of severe maternal morbidity.

### Limitations of the Review

This review included studies from developed and developing countries. As health care systems differ across countries, careful interpretation is required as findings from one country cannot be generalised to others. Studies were excluded if they did not include outcomes of severe maternal morbidity. However, some conditions, such as stillbirth and caesarean section, could be a consequence of severe maternal morbidity. As these are potential mediators or contributors to PTSD [66-68], excluding them might have limited understanding of the complexities of PTSD/PTSD symptoms following severe maternal morbidity. As we only included studies written in English, publication bias is a possibility, as positive findings are more likely to be published in English [69].

### Implications for practice

Despite the absence of robust evidence regarding the relationship between severe maternal morbidity and PTSD/PTSD symptoms, the results of our review suggest that maternal morbidity, particularly severe cases involving poor neonatal outcomes, may be followed by PTSD and its symptoms. It is crucially important that clinicians and policy makers are aware of possible PTSD symptoms in response to severe maternal morbidity since the incidence of severe maternal morbidity is increasing in many western countries [26,28]. Early and timely recognition of women at risk and appropriate referral is necessary, as this may reduce the duration of treatment [46,70] and potentially reduce subsequent long term burden of PTSD both to the individual and society [71].

### Further research

A comprehensive evaluation of the potential association between severe maternal morbidity and subsequent PTSD and PTSD symptoms is timely and important to inform the safety and quality of maternity care. There is an urgent need for prospective research with large sample sizes and appropriate recognition of important confounders. These studies will also require: 1) well-defined definitions of severe maternal morbidity; 2) sophisticated measurement of PTSD and its symptoms; and 3) inclusion of potential mediators or moderators (e.g., neonatal outcomes, subjective perception, recovery environment) in analysis to better understand mechanisms underlining the relationship between severe maternal morbidity and PTSD and PTSD symptoms. In addition, there is a need for greater openness and transparency in study reporting.

## Conclusion

The psychiatric impact of severe maternal morbidity remains uncertain, but this review suggests a potential relationship between severe maternal morbidity and PTSD and PTSD symptoms. Well-designed studies are necessary to understand the relationship and the mechanism underlying the association in order to minimise the longer term psychiatric impact of severe maternal morbidity. In line with NICE guidance [71], signs of PTSD symptoms following severe maternal morbidity should be monitored over time to make sure problems are identified as early as possible to enable timely, appropriate and effective care to meet individual needs to be implemented.

## Endnotes

^a^ Severe pre-eclampsia was defined as ‘pre-eclampsia and at least one of the following: severe blood pressure elevation defined by systolic blood pressure ≥ 160 mm Hg and/or diastolic blood pressure ≥ 110 mm Hg, severe proteinuria (5 or more grams in 24 h), HELLP syndrome defined by a thrombocyte count ≤ 100 × 109/l, and/or ASAT and ALAT above 30 U/l, eclamptic convulsions, or fetal growth restriction’ (p127) [42].

## Competing interests

The authors declare that they have no competing interests.

## Author's contributions

MF developed the review with the support of DB and JS. The search strategy was completed by MF, data extraction, analysis and methodological assessments were made by MF, DB and JS. All authors contributed to the review. All authors contributed to the manuscript. All authors read and approved the final manuscript.

## Pre-publication history

The pre-publication history for this paper can be accessed here:

http://www.biomedcentral.com/1471-2393/12/125/prepub

## Supplementary Material

Additional file 1 Prisma 2009 Cheklist. Click here for file

Additional file 2 Excluded studies and the reason for the exclusion. Click here for file

Additional file 3 Methodological quality of selected studies. Click here for file
