# Supplementary material for: A systematic review of the relationship between severe maternal morbidity and post-traumatic stress disorder
Source: BMC Pregnancy Childbirth. 2012 Nov 10;12:125. doi: 10.1186/1471-2393-12-125 (PMC3582425; doi:10.1186/1471-2393-12-125)
Supplement: Additional file 2 — Excluded studies and the reason for the exclusion. [file 1471-2393-12-125-S2.pdf]

Appendix 2

Excluded studies and the reason for the exclusion

No variable of maternal morbidity
AHLUND, S., CLARKE, P., HILL, J. & THALANGE, N. K. S. 2009. Post-traumatic stress symptoms in mothers of very low birth weight infants 2-3 years post-partum. *Archives of Womens Mental Health,* 12**,** 261-264.
ALLEN, S. F. C. 1996. *An investigation of post-traumatic stress disorder symptoms following traumatic labour experiences : causal factors, mediating variables and consequences. [electronic resource].* Thesis (Ph.D.), University of Southampton.
ANDERSON, C. & LOGAN, D. 2010. Impact of traumatic birth experience on Latina adolescent mothers. *Issues Ment Health Nurs,* 31**,** 700-7.
AYERS, S., HARRIS, R., SAWYER, A., PARFITT, Y. & FORD, E. 2009. *Posttraumatic stress disorder after childbirth: Analysis of symptom presentation and sampling*, Journal of Affective Disorders. 119 (1-3) (pp 200-204), 2009. Date of Publication: January 2009.
AYERS, S. & PICKERING, A. D. 2001. Do women get posttraumatic stress disorder as a result of childbirth? A prospective study of incidence. *Birth-Issues in Perinatal Care,* 28**,** 111-118.
BAILHAM, D. 2001. *Psychological trauma following childbirth. [electronic resource].* Thesis (Ph.D.), University of Warwick.
CZARNOCKA, J. & SLADE, P. 2000. Prevalence and predictors of post-traumatic stress symptoms following childbirth. *British Journal of Clinical Psychology,* 39**,** 35-51.
BECK, C. T., GABLE, R. K., SAKALA, C. & DECLERCQ, E. R. 2011. Posttraumatic Stress Disorder in New Mothers: Results from a Two-Stage U.S. National Survey. Birth, 38, 216-227.
DAVIES, J., SLADE, P., WRIGHT, I. & STEWART, P. 2008. *Posttraumatic stress symptoms following childbirth and mothers' perceptions of their infants*, Infant Mental Health Journal. 29 (6) (pp 537-554), 2008. Date of Publication: 2008.
DAVIS, L., EDWARDS, H., MOHAY, H. & WOLLIN, J. 2003. The impact of very premature birth on the psychological health of mothers. *Early Human Development,* 73**,** 61-70.
DEMIER, R. L., HYNAN, M. T., HARRIS, H. B. & MANNIELLO, R. L. 1996. Perinatal stressors as predictors of symptoms of posttraumatic stress in mothers of infants at high risk. *Journal of Perinatology,* 16**,** 276-80.
DENIS, A., PARANT, O. & CALLAHAN, S. 2011. *Post-traumatic stress disorder related to birth: A prospective longitudinal study in a French population*, Journal of Reproductive and Infant Psychology. 29 (2) (pp 125-135), 2011. Date of Publication: April 2011.
ELHAI, J. D., FRUEH, B. C., GOLD, P. B., HAMNER, M. B. & GOLD, S. N. 2003. Posttraumatic Stress, Depression and Dissociation as Predictors of MMPI-2 Scale 8 Scores in Combat Veterans with PTSD. *Journal of Trauma & Dissociation,* 4**,** 51-64.
ELKLIT, A., HARTVIG, T. & CHRISTIANSEN, M. 2007. *Psychological sequelae in parents of extreme low and very low birth weight infants*, Journal of Clinical Psychology in Medical Settings. 14 (3) (pp 238-247), 2007. Date of Publication: Sep 2007.
FAIRBROTHER, N. & WOODY, S. R. 2007. Fear of childbirth and obstetrical events as predictors of postnatal symptoms of depression and post-traumatic stress disorder. *Journal of Psychosomatic Obstetrics & Gynecology,* 28**,** 239-42.
FEELEY, N., ZELKOWITZ, P., CORMIER, C., CHARBONNEAU, L., LACROIX, A. & PAPAGEORGIOU, A. 2011. Posttraumatic stress among mothers of very low birthweight infants at 6 months after discharge from the neonatal intensive care unit. *Applied Nursing Research,* 24**,** 114-7.
FORD, E., AYERS, S. & BRADLEY, R. 2010. Exploration of a cognitive model to predict post-traumatic stress symptoms following childbirth. *Journal of Anxiety Disorders,* 24**,** 353-9.
GAMBLE, J., CREEDY, D., MOYLE, W., WEBSTER, J., MCALLISTER, M. & DICKSON, P. 2005. Effectiveness of a counseling intervention after a traumatic childbirth: a randomized controlled trial. *Birth (Berkeley, Calif.)* [Online]. Available:http://www.mrw.interscience.wiley.com/cochrane/clcentral/articles/141/CN-00513141/frame.html.
HOLDITCH-DAVIS, D., BARTLETT, T. R., BLICKMAN, A. L. & MILES, M. S. 2003. *Posttraumatic stress symptoms in mothers of premature infants*, Journal of obstetric, gynecologic, and neonatal nursing : JOGNN / NAACOG. 32 (2) (pp 161-171), 2003. Date of Publication: 2003 Mar-Apr.
ILES, J., SLADE, P. & SPIBY, H. 2011. *Posttraumatic stress symptoms and postpartum depression in couples after childbirth: The role of partner support and attachment*, Journal of Anxiety Disorders. 25 (4) (pp 520-530), 2011. Date of Publication: May 2011.
KEOGH, E., AYERS, S. & FRANCIS, H. 2002. Does anxiety sensitivity predict post-traumatic stress symptoms following childbirth? A preliminary report. *Cognitive Behaviour Therapy,* 31**,** 145-155.
KERSTING, A., KROKER, K., STEINHARD, J., HOERNIG-FRANZ, I., WESSELMANN, U., LUEDORFF, K., OHRMANN, P., AROLT, V. & SUSLOW, T. 2009. *Psychological impact on women after second and third trimester termination of pregnancy due to fetal anomalies versus women after preterm birth-a 14-month follow up study*, Archives of Women's Mental Health. 12 (4) (pp 193-201), 2009. Date of Publication: August 2009.
LEEDS, L. & HARGREAVES, I. 2008. *The psychological consequences of childbirth*, Journal of Reproductive and Infant Psychology. 26 (2) (pp 108-122), 2008. Date of Publication: May 2008.
LEFKOWITZ, D. S., BAXT, C. & EVANS, J. R. 2010. Prevalence and correlates of posttraumatic stress and postpartum depression in parents of infants in the Neonatal Intensive Care Unit (NICU). *Journal of Clinical Psychology in Medical Settings,* 17**,** 230-7.
LYONS, S. 1998. *A prospective study of post traumatic stress symptoms 1 month following childbirth in a group of 42 first-time mothers*, Journal of Reproductive and Infant Psychology. 16 (2-3) (pp 91-105), 1998. Date of Publication: 1998.
MENAGE, J. 1993. Post-traumatic stress disorder in women who have undergone obstetric and/or gynaecological procedures. *Journal of Reproductive and Infant Psychology,* 11**,** 221-228.
ONOYE, J. M., GOEBERT, D., MORLAND, L., MATSU, C. & WRIGHT, T. 2009. PTSD and postpartum mental health in a sample of Caucasian, Asian, and Pacific Islander women. *Archives of Women's Mental Health,* 12**,** 393-400.
PRIEST, S. R., HENDERSON, J., EVANS, S. F. & HAGAN, R. 2003. Stress debriefing after childbirth: a randomised controlled trial. *The Medical journal of Australia* [Online]. Available: http://www.mrw.interscience.wiley.com/cochrane/clcentral/articles/738/CN-00437738/frame.html.

SALVESEN, K. A., OYEN, L., SCHMIDT, N., MALT, U. F. & EIK-NES, S. H. 1997. Comparison of long-term psychological responses of women after pregnancy termination due to fetal anomalies and after perinatal loss. *Ultrasound in Obstetrics & Gynecology,* 9**,** 80-5.

SELKIRK, R., MCLAREN, S., OLLERENSHAW, A., MCLACHLAN, A. J. & MOTEN, J. 2006. *The longitudinal effects of midwife-led postnatal debriefing on the psychological health of mothers*, Journal of Reproductive and Infant Psychology. 24 (2) (pp 133-147), 2006. Date of Publication: May 2006.

SKARI, H., MALT, U. F., BJORNLAND, K., EGELAND, T., HAUGEN, G., SKREDEN, M., DALHOLT BJORK, M., BJORNSTAD OSTENSEN, A. & EMBLEM, R. 2006. Prenatal diagnosis of congenital malformations and parental psychological distress--a prospective longitudinal cohort study. *Prenat Diagn,* 26**,** 1001-9.

SKARI, H., SKREDEN, M., MALT, U. F., DALHOLT, M., OSTENSEN, A. B., EGELAND, T. & EMBLEM, R. 2002. Comparative levels of psychological distress, stress symptoms, depression and anxiety after childbirth - A prospective population-based study of mothers and fathers.

SODERQUIST, J., WIJMA, B., THORBERT, G. & WIJMA, K. 2009. Risk factors in pregnancy for post-traumatic stress and depression after childbirth. *BJOG: An International Journal of Obstetrics & Gynaecology,* 116**,** 672-80.

SODERQUIST, J., WIJMA, B. & WIJMA, K. 2006. The longitudinal course of post-traumatic stress after childbirth. *Journal of Psychosomatic Obstetrics & Gynecology,* 27**,** 113-9.

SODERQUIST, J., WIJMA, K. & WIJMA, B. 2002. Traumatic stress after childbirth: The role of obstetric variables.

SOET, J. E., BRACK, G. A. & DIIORIO, C. 2003. Prevalence and predictors of women's experience of psychological trauma during childbirth. *Birth,* 30**,** 36-46.

VAN SON, M. J. M., VERKERK, G., VAN DER HART, O., KOMPROE, I. & POP, V. 2005. Prenatal depression, mode of delivery and perinatal dissociation as predictors of postpartum posttraumatic stress: An empirical study.

VANDERBILT, D., BUSHLEY, T., YOUNG, R. & FRANK, D. A. 2009. Acute posttraumatic stress symptoms among urban mothers with newborns in the neonatal intensive care unit: a preliminary study. *Journal of Developmental & Behavioral Pediatrics,* 30**,** 50-6.

WENZEL, A., HAUGEN, E. N., JACKSON, L. C. & BRENDLE, J. R. 2005. Anxiety symptoms and disorders at eight weeks postpartum. *Journal of Anxiety Disorders,* 19**,** 295-311.

WHITE, T., MATTHEY, S., BOYD, K. & BARNETT, B. 2006. *Postnatal depression and post-traumatic stress after childbirth: Prevalence, course and co-occurrence*, Journal of Reproductive and Infant Psychology. 24 (2) (pp 107-120), 2006. Date of Publication: May 2006.

WIJMA, K., SODERQUIST, J. & WIJMA, B. 1997a. Posttraumatic stress disorder after childbirth: a cross sectional study. *Journal of Anxiety Disorders,* 11**,** 587-97.

ZAERS, S., WASCHKE, M. & EHLERT, U. 2008. Depressive symptoms and symptoms of post-traumatic stress disorder in women after childbirth. *Journal of Psychosomatic Obstetrics & Gynecology,* 29**,** 61-71.

ZANARDO, V., GAMBINA, I., BEGLEY, C., LITTA, P., COSMI, E., GIUSTARDI, A. & TREVISANUTO, D. 2011. Psychological distress and early lactation performance in mothers of late preterm infants. *Early Human Development,* 87**,** 321-3.

Assessed different dimensions of psychological issues

ANDERSGAARD, A. B., HERBST, A., JOHANSEN, M., BORGSTROM, A., BILLE, A. G. & OIAN, P. 2009. Follow-up interviews after eclampsia.

FILIPPI, V., GOUFODJI, S., SISMANIDIS, C., KANHONOU, L., FOTTRELL, E., RONSMANS, C., ALIHONOU, E. & PATEL, V. 2010. Effects of severe obstetric complications on women's health and infant mortality in Benin. *Tropical Medicine & International Health,* 15**,** 733-742.

FOTTRELL, E., KANHONOU, L., GOUFODJI, S., BEHAGUE, D. P., MARSHALL, T., PATEL, V. & FILIPPI, V. 2010. Risk of psychological distress following severe obstetric complications in Benin: the role of economics, physical health and spousal abuse. *British Journal of Psychiatry,* 196**,** 18-25.

LEENERS, B., STILLER, R., NEUMAIER-WAGNER, P., KUSE, S., SCHMITT, A. & RATH, W. 2008. Psychosocial distress associated with treatment of hypertensive diseases in pregnancy. *Psychosomatics,* 49**,** 413-419.

REP, A., GANZEVOORT, W., BONSEL, G. J., HANS, W. & DE VRIES, J. I. P. 2007. Psychosocial impact of early-onset hypertensive disorders and related complications in pregnancy. *American Journal of Obstetrics and Gynecology,* 197.

ROES, E. M., RAIJMAKERS, M. T. M., SCHOONENBERG, M., WANNER, N., PETERS, W. H. M. & STEEGERS, E. A. P. 2005. Physical well-being in women with a history of severe preeclampsia. Journal of Maternal-Fetal & Neonatal Medicine, 18, 39-45.

UKPONG, D. I. 2011. Factors associated with psychological morbidity in mothers of pre-term infants: a study from Wesley Guild Hospital, Nigeria. *Journal of Obstetrics & Gynaecology,* 31**,** 146-8.

WHITE, O., MCCORRY, N. K., SCOTT-HEYES, G., DEMPSTER, M. & MANDERSON, J. 2008. Maternal appraisals of risk, coping and prenatal attachment among women hospitalised with pregnancy complications. *Journal of Reproductive and Infant Psychology,* 26**,** 74-85.

Maternal morbidity clustered together with other independent variables and not analysed separately

CIGOLI, V., GILLI, G. & SAITA, E. 2006. Relational factors in psychopathological responses to childbirth. *Journal of Psychosomatic Obstetrics & Gynecology,* 27**,** 91-7.

MAGGIONI, C., MARGOLA, D. & FILIPPI, F. 2006. PTSD, risk factors, and expectations among women having a baby: a two-wave longitudinal study. *Journal of Psychosomatic Obstetrics & Gynecology,* 27**,** 81-90.

No data of maternal morbidity

LEMOLA, S., STADLMAYR, W. & GROB, A. 2007. *Maternal adjustment five months after birth: The impact of the subjective experience of childbirth and emotional support from the partner*, Journal of Reproductive and Infant Psychology. 25 (3) (pp 190-202), 2007. Date of Publication: Aug 2007.

No assessment of the association between maternal morbidity and subsequent PTSD/PTSD symptoms
FORD, E. 2008. *The role of support and control during birth in the development of post-traumatic stress disorder following childbirth.* Thesis (D. Phil.) 1 v. ; 31 cm., University of Sussex.<br>POEL, Y. H. M., SWINKELS, P. & DE VRIES, J. I. P. 2009. Psychological treatment of women with psychological complaints after pre-eclampsia. *Journal of Psychosomatic Obstetrics & Gynecology,* 30**,** 65-72.<br>RYDING, E. L., WIJMA, B. & WIJMA, K. 1997. Posttraumatic stress reactions after emergency cesarean section. *Acta Obstetricia et Gynecologica Scandinavica,* 76**,** 856-61.<br>THOMPSON, J. F., ROBERTS, C. L. & ELLWOOD, D. A. 2011. Emotional and physical health outcomes after significant primary post-partum haemorrhage (PPH): A multicentre cohort study. Australian & New Zealand Journal of Obstetrics & Gynaecology, 51, 365-371.
**Letter/commentary/news/short communications**
GOLDBECK-WOOD, S. 1996. Post-traumatic stress disorder may follow childbirth. *BMJ,* 313**,** 774.<br>GRIEBENOW, J. J. 2006. Healing the trauma: entering motherhood with posttraumatic stress disorder (PTSD). *Midwifery Today with International Midwife***,** 28-31.<br>LOVETT, K. F. 2001. PTSD and stillbirth. *British Journal of Psychiatry,* 179**,** 367; author reply 368.<br>MCKENZIE-MCHARG, K. 2004. Traumatic birth: understanding predictors, triggers, and counseling process is essential to treatment. *Birth,* 31**,** 219-21.<br>SHARMA, J. B., NEWMAN, M. R. & SMITH, R. J. 1993. Psychological distress and preterm delivery. Consider urogenital infection. *BMJ,* 307**,** 934.<br>SHEEHAN, J. D. 2001. PTSD and stillbirth. *British Journal of Psychiatry,* 179**,** 368; author reply 368.<br>VYTHILINGUM, B. 2010. *Should childbirth be considered a stressor sufficient to meet the criteria for PTSD?*, Archives of Women's Mental Health. 13 (1) (pp 49-50), 2010. Date of Publication: February 2010.
**Assessed PTSD in pregnancy**
SENG, J. S., LOW, L. K., SPERLICH, M., RONIS, D. L. & LIBERZON, I. 2009. Prevalence, Trauma History, and Risk for Posttraumatic Stress Disorder Among Nulliparous Women in Maternity Care. *Obstetrics and Gynecology,* 114**,** 839-847.<br>SENG, J. S., OAKLEY, D. J., SAMPSELLE, C. M., KILLION, C., GRAHAM-BERMANN, S. & LIBERZON, I. 2001. *Posttraumatic stress disorder and pregnancy complications*, Obstetrics and Gynecology. 97 (1) (pp 17-22), 2001. Date of Publication: 2001.
**Qualitative studies or case reports**
BALLARD, C. G., STANLEY, A. K. & BROCKINGTON, I. F. 1995. Post-traumatic stress disorder (PTSD) after childbirth. *British Journal of Psychiatry,* 166**,** 525-8.<br>CARTY, S. 2007. *Trauma following childbirth [electronic resource].* Thesis (Clin.Psy.D), University of Birmingham 2007.<br>FONES, C. 1996. Posttraumatic stress disorder occurring after painful childbirth. *Journal of Nervous & Mental Disease,* 184**,** 195-6.<br>VAN PAMPUS, M. G., WOLF, H., WEIJMAR SCHULTZ, W. C. M., NEELEMAN, J. & AARNOUDSE, J. G. 2004. Posttraumatic stress disorder following pre-eclampsia and HELLP syndrome. *Journal of Psychosomatic Obstetrics & Gynecology,* 25**,** 183-7.
**Irrelevant population**
COOPER, R., MISHRA, G., HARDY, R. & KUH, D. 2009. Hysterectomy and subsequent psychological health: Findings from a British birth cohort study. *Journal of Affective Disorders,* 115**,** 122-130.<br>KHASTGIR, G., STUDD, J. W. W. & CATALAN, J. 2000. The psychological outcome of hysterectomy. *Gynecological Endocrinology,* 14**,** 132-141.<br>YEN, J.-Y., CHEN, Y.-H., LONG, C.-Y., CHANG, Y., YEN, C.-F., CHEN, C.-C. & KO, C.-H. 2008. Risk factors for major depressive disorder and the psychological impact of hysterectomy: A prospective investigation. *Psychosomatics,* 49**,** 137-142.
**Reviews or review protocols that did not look at the association between maternal morbidity and PTSD/PTSD symptoms**
ALDER, J., STADLMAYR, W., TSCHUDIN, S. & BITZER, J. 2006. Post-traumatic symptoms after childbirth: what should we offer? *Journal of Psychosomatic Obstetrics & Gynecology,* 27**,** 107-12.<br>ANDERSEN, L. B., MELVAER, L. B., VIDEBECH, P., LAMONT, R. F. & JOERGENSEN, J. S. 2012. Risk factors for developing post-traumatic stress disorder following childbirth: A systematic review. Acta Obstetricia et Gynecologica Scandinavica, 1-28.<br>AYERS, S. 2004. Delivery as a traumatic event: prevalence, risk factors, and treatment for postnatal posttraumatic stress disorder. *Clinical Obstetrics & Gynecology,* 47**,** 552-67.<br>BASTOS, M. H., BICK, D., ROWAN, C. J., SMALL, R. & MCKENZIE-MCHARG, K. 2008. Debriefing for the prevention of psychological trauma in women following childbirth. *Cochrane Database of Systematic Reviews* [Online]. Available: http://www.mrw.interscience.wiley.com/cochrane/clsysrev/articles/CD007194/frame.html.<br>BICK, D. & ROWAN, C. 2007. Postnatal maternal mental health: an update on depression and post-traumatic stress disorder following birth. *Journal of the Association of Chartered Physiotherapists in Women's Health***,** 4-13.<br>CLEMENT, S. 2001. Psychological aspects of caesarean section. *Best Practice & Research in Clinical Obstetrics & Gynaecology,* 15**,** 109-26.<br>CUMBERBATCH, C.-J., BIRNDORF, C. & DRESNER, N. 2005. Psychological implications of high-risk pregnancy. *International Journal of Fertility & Womens Medicine,* 50**,** 180-6.<br>GRIEBENOW, J. J. 2006. Healing the trauma: entering motherhood with posttraumatic stress disorder (PTSD). *Midwifery Today with International Midwife***,** 28-31.<br>OLDE, E., VAN DER HART, O., KLEBER, R. & VAN SON, M. 2006. Posttraumatic stress following childbirth: A review. *Clinical Psychology Review,* 26**,** 1-16.<br>REYNOLDS, J. L. 1997. Post-traumatic stress disorder after childbirth: the phenomenon of traumatic birth. CMAJ Canadian Medical Association Journal, 156, 831-5.<br>ROSS, L. E. & MCLEAN, L. M. 2006. Anxiety disorders during pregnancy and the postpartum period: A systematic review. *Journal of Clinical Psychiatry,* 67**,** 1285-98.

SAWYER, A., AYERS, S. & SMITH, H. 2010. Pre- and postnatal psychological wellbeing in Africa: a systematic review. *Journal of Affective Disorders,* 123**,** 17-29.
STONE, H. L. 2009. Post-traumatic stress disorder in postpartum patients: what nurses can do. *Nursing for Women's Health,* 13**,** 284-91.
TEDSTONE, J. E. & TARRIER, N. 2003. Posttraumatic stress disorder following medical illness and treatment. *Clinical Psychology Review,* 23**,** 409-48.

The same study (published or non-published)

CREEDY, D. K., SHOCHET, I. M. & HORSFALL, J. 2000. Childbirth and the development of acute trauma symptoms: Incidence and contributing factors. *Birth-Issues in Perinatal Care,* 27**,** 104-111.
