# Supplementary material for: A systematic review of the relationship between severe maternal morbidity and post-traumatic stress disorder
Source: BMC Pregnancy Childbirth. 2012 Nov 10;12:125. doi: 10.1186/1471-2393-12-125 (PMC3582425; doi:10.1186/1471-2393-12-125)
Supplement: Additional file 3 — Methodological quality of selected studies. [file 1471-2393-12-125-S3.doc]

**Appendix**

Appendix 3: Methodological quality of selected studies

|  | **Adewuya** **et.al.** **2006** | **Ayers** **1999** | **Baecke** **et.al.** **2009** | **Cohen** **et.al.** **2004** | **Creedy** **1999** | **Engelhard** **et.al.** **2002** | **Hoedjes** **et** **al.** **2001** | **Lev-Wiesel** **et** **al.** **2009** | **Sorenson** **&** **Tschetter** **2010** | **Stramrood** **et** **al.** **2010** | **Stramrood** **et** **al.** **2011** |
| --- | --- | --- | --- | --- | --- | --- | --- | --- | --- | --- | --- |
| **Was** **the** **cohort** **recruited** **in** **an** **acceptable** **way** **(to** **assess** **the** **association** **between** **PTSD** **(symptoms)** **and** **severe** **maternal** **morbidity** **(SMM)?** | Yes | Unclear - excluded women with elective caesarean section | Unclear *-* *recruitment* *process:* *uncertain* | Unclear *-* *excluded* *women* *with* *risk* *of* *baby* | Unclear *-* *excluded* *women* *with* *medical* *risk* | Unclear *-* *recruited* *only* *primiparas* | Unclear *-* *recruited* *only* *women* *with* *preeclampsia* | Unclear *-* *recruitment* *process:* *uncertain* | Unclear *-* *recruited* *women* *using* *newspaper* *and* *public* *phone* *book* | Unclear *-* *excluded* *multiple* *pregnancy* *etc.* | Yes |
|  |  |  |  |  |  |  | *-* *no* *healthy* *control* |  |  |  |  |
| ***(sample*** ***representativeness)*** |  |  | *-* *small* *sample* *^†^* | *-* *small* *sample* *^†^* |  | *-* *small* *sample* *^†^* | *-* *small* *sample* *^†^* |  | *-* *small* *sample* *^†^* | *-* *small* *sample* *^†^* |  |
| **Was** **the** **SMM** **accurately** **measured** **to** **minimize** **bias?** | Unclear *hospital* *admission:* *reason* *uncertain* | Unclear *-* *combined* *different* *types* *of* *complications* *with* *less* *severe* *cases* *^‡^* | Yes | Unclear *-* *combined* *different* *types* *of* *complications* *with* *less* *severe* *cases* *^‡^* | Unclear *-* *combined* *different* *types* *of* *complications* *with* *less* *severe* *cases* *^‡^* | Yes | Yes | Unclear *-* *combined* *different* *types* *of* *complications* *with* *less* *severe* *cases* *^‡^* | Unclear *-* *definition,* *type* *and* *data* *source* *of* *complication:* *uncertain* *^‡^* | Yes | Unclear *-* *data* *source:* *self-report* *^‡^* |
|  | *-* *data* *source:* *self-report* *^‡^* |  |  | *-* *data* *source:* *uncertain* *^‡^* | *-* *data* *source:* *self-report* *^‡^* |  |  | *-* *data* *source:* *partially* *self-report* *^‡^* |  |  |  |
| **Were** **PTSD** **or** **PTSD** **symptoms** **accurately** **measured** **to** **minimize** **bias?** | Yes | Yes | Yes | Yes | Yes | Yes | Yes | Yes | Unclear *-* *scale:* *validity* *not* *established* *^‡^* | Yes | Yes |
| **Have** **the** **authors** **identified** **all** **important** **confounding** **factors** | Yes | Yes | No *-* *pre-existing* *psychological* *issues:* *not* *mentioned* | Yes | Yes | No *-* *pre-existing* *psychological* *issues:* *not* *mentioned* | Yes | Yes | No - pre-existing psychological condition: not mentioned | Yes | Yes |
| **Have** **they** **taken** **account** **of** **the** **confounding** **factors** **in** **the** **design** **and/or** **analysis?** | Yes | No *-* *pre-existing* *PTSD* *was* *controlled* *for,* *but* *not* *others* | No *-* *numbers* *of* *pre-existing* *psychological* *issues:* *not* *controlled* *for* | Yes, except for controlling for pre-existing PTSD | Yes, except for controlling for pre-existing PTSD | No *-* *numbers* *of* *pre-existing* *psychological* *issues:* *not* *controlled* *for* | No *-* *assessment* *time* *was* *controlled* *for,* *but* *not* *others* | Yes | No *-* *unadjusted* *analysis* | Yes | Yes, except for controlling for pre-existing PTSD |
| **Was** **the** **follow** **up** **of** **subjects** **complete** **enough?** **(eg.** **the** **persons** **that** **are** **lost** **to** **follow-up** **may** **have** **different** **outcomes** **than** **those** **available** **for** **assessment)** | Yes | Yes | Unclear | Yes | Yes | Yes | Yes | Unclear | Unclear | Unclear | Unclear |
|  |  | *-* *non-response* *bias* *clearly* *discussed* | *-* *non-response* *bias:* *not* *discussed* *^‡^* | *-* *non-response* *bias* *clearly* *discussed* | *-* *non-response* *bias* *clearly* *discussed* | *-* *non-response* *bias* *clearly* *discussed* | *-* *non-response* *bias* *clearly* *discussed* | *-* *small* *dropout* *rate,* *but* *women* *who* *did* *not* *consent* *at* *the* *recruitment* *was* *not* *reported* *^‡^* | *-* *non-response* *bias:* *not* *discussed* *^‡^* | *-* *women* *not* *willing* *to* *participate* *was* *not* *reported* *^‡^* | *-* *non-response* *bias:* *not* *assessed* *^‡^* |
| **Was** **the** **follow** **up** **of** **subjects** **long** **enough?** | Yes | Yes | Yes | Yes | Yes | Yes | Yes | Yes | Yes | Yes | Yes |
| **Other** **limitations** | Cross-sectional | -- | -- | -- | -- | Retrospectively collected key variables up to previous 2 years^‡^ | -- | -- | cross-sectional | -- | Cross-sectional |

Note: ‡ There is a possibility of information bias due to misdiagnosis, recall bias or missing data (ie. refusals, non-participation, non-response). † There is a possibility of low statistical power.
